# First results from the mustatils of the Harrat Khaybar: New insights into neolithic cult in Northwest Saudi Arabia

**DOI:** 10.1371/journal.pone.0358444

**Published:** 2026-09-18

**Authors:** Melissa Kennedy, Laura Strolin, Jane McMahon, Anas Abahussain, Adel Alqaibari, Naif Alshehri, Hugh Thomas

**Affiliations:** 1 Archaeology, University of Sydney Australia, Sydney, Australia; 2 Classics and Ancient History, University of Western Australia, Perth, Australia; 3 Vere Gordon Childe Centre, The University of Sydney, Sydney, Australia; 4 Museum of Natural History of Geneva, Geneva, Switzerland; 5 Archaeology Stable Isotope Laboratory, University of Kiel, Kiel, Germany; 6 Institute for Prehistoric and Protohistoric Archaeology, University of Kiel, Kiel, Germany; 7 The Heritage Commission, Ministry of Culture, Riyadh, Saudi Arabia; Instititut Català de Paleoecologia Humana i Evolució Social (IPHES-CERCA), SPAIN

## Abstract

In 2023, the University of Sydney, in partnership with the Heritage Commission undertook research at a large mustatil site to the southeast of Al-Thamad on the Harrat Khaybar, Saudi Arabia. This site is marked by a concentration of 28 mustatils, as well as a series of other monumental stone-built features, dating from the Neolithic onwards. These are the first mustatils to be investigated on the Harrat Khaybar, where more than 400 have been identified. During fieldwork, all mustatils at the site were documented, with test excavations conducted in three structures. Dateable surface material was also obtained from several other mustatils at the site. The results of these test excavations and surface sampling sheds new light on and gives important insight into the mustatil tradition. Based on this dataset, and the comparative material available from AlUla County, it would appear that the mustatil tradition may have been a cohesive ritual concept extending across a large part of Saudi Arabia, potentially making it one of the earliest, widespread ritual traditions known to date.

## Introduction

Over the last five years, extensive surveys and excavations have provided the first comprehensive understanding of mustatils (مستطيل, the Arabic for ‘rectangle’), identifying more than 1600 of these monumental Neolithic structures across northern Arabia [1–6]. Excavations in the county of AlUla, northwest Saudi Arabia, have dated these structures to the late 6^th^ through to early 5^th^ millennia BCE [3–6]. Based on this work, a cultic/ritual function has been ascribed to these structures due to their monumental scale, their highly distinctive architectural features, and the carefully selected and prepared faunal elements found within the chambers of each excavated mustatil. These deposits consisted of upper cranial elements of mainly domestic, but also wild horned ruminants. Assemblages are composed overwhelmingly of domestic cattle and *Bos* sp. remains, with variable quantities of domestic caprines and wild ruminants [4,6]. Radiocarbon assays also suggest a somewhat restricted date range between ca. 5300−4700 BCE for the phenomenon [6]. With the date range and species breakdown of the AlUla mustatils now broadly understood, this brings to the fore the date and composition of the remainder of northern Arabia’s mustatils. As such, in late 2023, the University of Sydney’s *Prehistoric AlUla and Khaybar Excavation Project*, in conjunction with the Heritage Commission, Ministry of Culture, undertook the first archaeological work on the mustatils of the Harrat Khaybar, at an area to the south of the town of Al-Thamad. This area has some of the densest concentrations of mustatils in northern Arabia. This paper will present the results of this new work and will provide the first excavation data for the mustatils outside of AlUla County, presenting important contextual chrono-cultural information for the wider mustatil tradition across northern Arabia.

## Methods

### Study area

**Fig 1:**
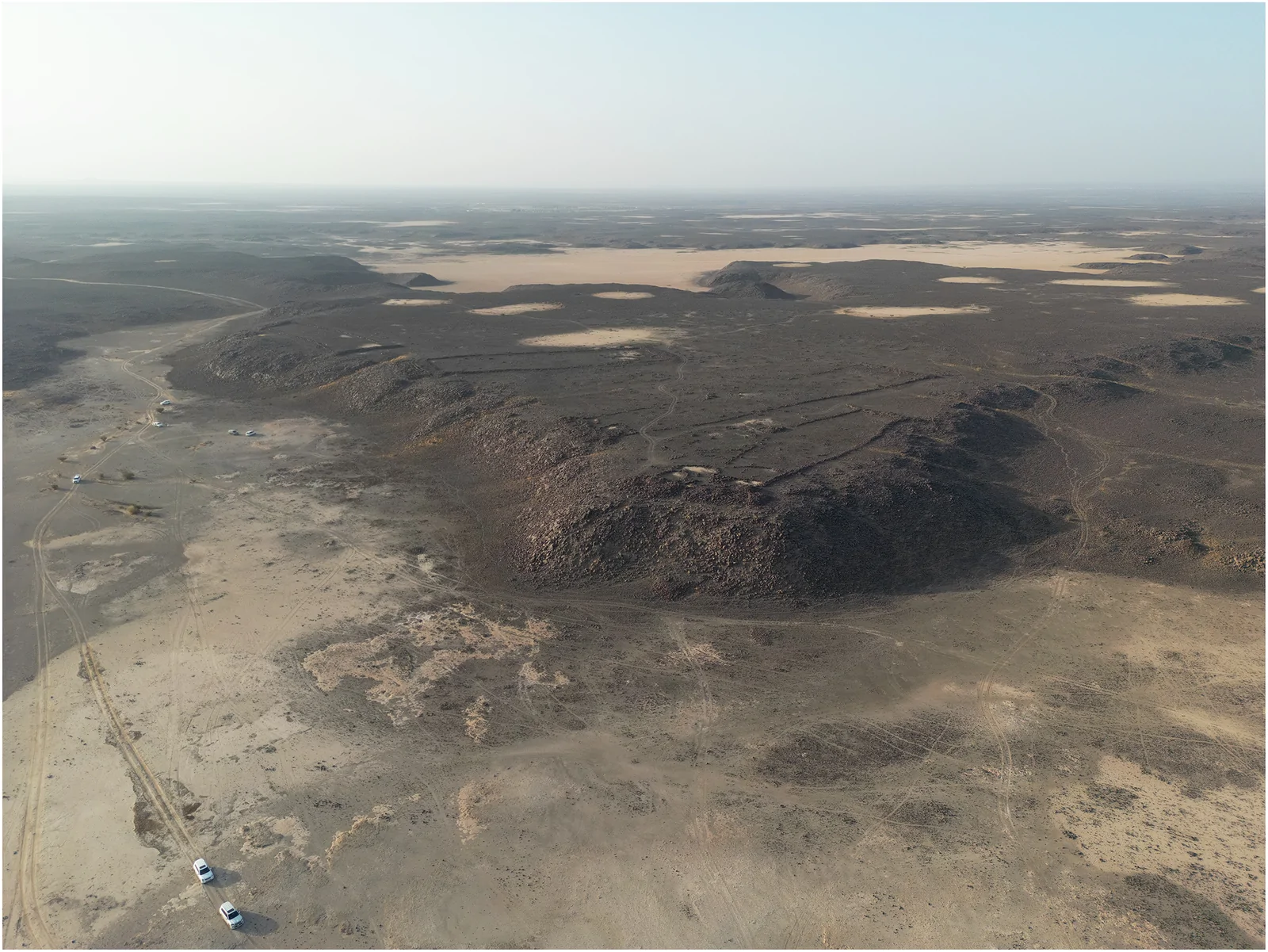
Oblique aerial view of the study area, notice the qa’ surrounding the site, photo orientated to the south.

The study area is located on the Harrat Khaybar. The Harrat Khaybar is a large volcanic area (*harrah*, حرة, lava fields), covering some 14,064 of km ^2^ of the Madinah Province, Saudi Arabia. The region is dominated by a 100 km long linear (north-south) vent system, with lava flows dating from as early as 5 MYA through to the Holocene [7]. To date, tens of thousands of archaeological sites and features are identifiable across this basalt landscape [8]. The study site lies approximately 7.5 km to the south-east of the modern town of Al-Thamad. The area is characterised by basalt plateaux that surround a series of alluvial depressions (singular *qa’,* قاع) that hold water after rain (Fig 1). Almost all investigated archaeological features were located on the plateaux above these alluvial depressions.

All necessary permits were obtained for the described study, which complied with all relevant regulations.

### Survey method

**Fig 2:**
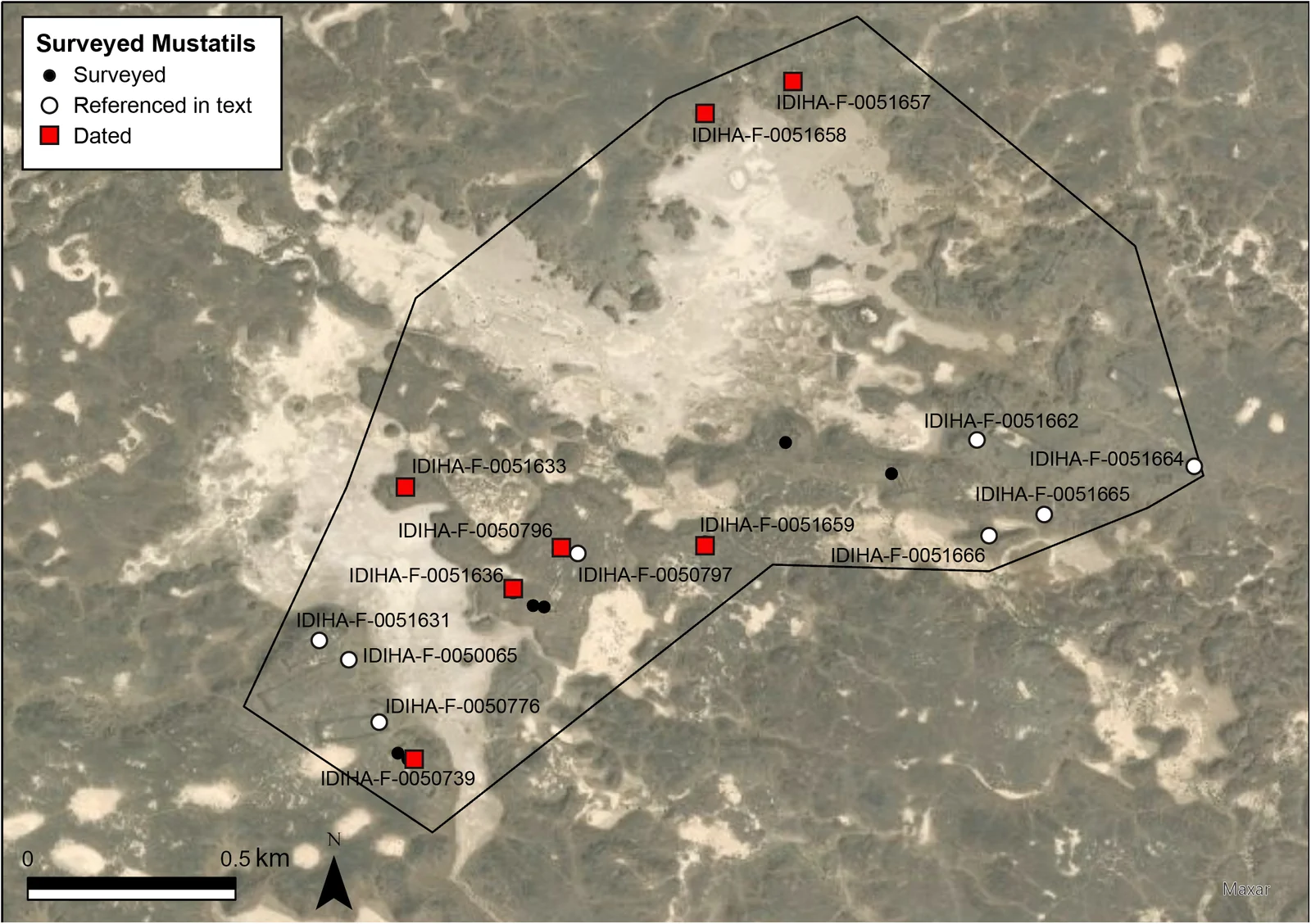
Map of mustatils excavated and surveyed at the study site. Basemap source: Esri, Maxar, Earthstar Geographics, and the GIS User Community.

This area was chosen for analysis due to it’s high concentration of mustatils, 28 in total (including seven I-type platforms), as well as other monumental structures, such as walls, tombs, and mounds. Work in this area took the form of both pedestrian ground survey and excavation, with all 28 mustatils surveyed, with test excavations conducted in three mustatils (Fig 2). On site recording included: detailed photography, drone-based and terrestrial photogrammetry along with written notes. These notes included information such as feature/typological form, construction technique and materials, geometry and preservation. Measurements of the x, y and z axes were recorded where possible, but precise measurements were not able to be obtained for some facets of these structures due to collapse and rocky debris. Transects were walked across all investigated structures.

### Excavation method

For the excavation component of the project, single context recording was utilised. This included context sheets and diary recording for each excavated structure and context. Recorded details included information regarding sediment colour and consistency; depth and extent; inclusions; spatial (stratigraphic) relationships; bioturbation/disturbance; as well as plans/drawings of each context, relevant sections, Harris matrices, and any other facets deemed of note. Contexts were excavated stratigraphically when changes in soil colour and consistency were identifiable. It should be noted that the term ‘context’ and ‘stratigraphic phase’ are distinct concepts in the following discussion. Although it can be argued that each ‘context’ represents a unique stratigraphic phase, multiple ‘contexts’ can relate to a single broader chrono-stratigraphic phase. All features were excavated by hand, with all sediment sieved through a 2 mm or 5 mm mesh. A 100% collection methodology was utilised. A detailed description of our excavation methodology has been previously published [5,9]. It should also be noted that although sediment samples were collected for flotation, no archaeobotanical remains were recovered. Finally, due inclement weather, which prohibited site access for a number of days, the overall excavation plan had to be significantly modified, with work focusing only on the mustatil chambers. This methodology targeted the recovery dating material and information on the assemblage and function of each of the excavation targets to facilitate regional comparison with excavated mustatils in AlUla. Excavations were not conducted within the courtyards or base, as initially planned, due to these constraints. However, little depositional material was present in the courtyards, with outcropping bedrock present throughout each of the excavated structures. However, it should also be noted that excavations within the courtyards of mustatils in AlUla have not revealed any associated finds. As such, the precise function of the courtyards themselves cannot be ascertained, although they must have played a key part in the ritual activity undertaken within the broader structure.

All finds (specimens) recovered during this fieldwork are housed in the AlUla Museum, AlUla, Saudi Arabia with the PAKEP archive.

### Faunal analysis

Faunal remains were recovered through both excavation and surface collection. Faunal remains from excavated contexts were recovered through sieving, whilst all visible surface material within the chambers in the heads was collected by hand. Bones were dry brushed before observation. Faunal identification is based on osteological morphology by means of comparative osteological data-sets (the osteological collection of the Museum of Natural History of Geneva (MNHG) and modern local specimens), as well as through specialized literature [10,11]. In the case of bovines, when it was not possible to discriminate between domestic and wild taxa, the term *Bos* sp. is used for remains that may belong to *Bos taurus* or *Bos primigenius*. The term large ungulate is used when, due to fragmentation, it was not possible to exclude equids and camel. The term indetermined caprines is used for undistinguished *Capra hircus*, *Ovis aries* and *Capra ibex nubiana*, with the term small ruminants used when gazelle could not be excluded.

## Results

The mustatils of Saudi Arabia are generally marked by a rectangular to sub-rectangular form and are marked by several key components: head and base platforms, a courtyard delimited by long walls between the head and base platforms, and in some instances associated features, such as circular stone cells and orthostats [3]. A narrow entranceway is located in the base platform, while the head contains one or more chambers where ritual offerings were deposited. However, the mustatils of the Harrat Khaybar are marked by greater variation of architectural form and complexity in the base and the associated features than those recorded in AlUla and elsewhere across northern Arabia. These variations are not readily identifiable in satellite imagery and can only be clearly seen through low-level aerial reconnaissance or on the ground. Four distinct mustatil variants were identified in the survey area. Variants 2, 3 and 4 had not previously been documented in AlUla, whilst the remainder conform to the standard mustatil form outlined in Thomas *et al.* [3]. The variants are as follows:

### Variant 1

**Fig 3:**
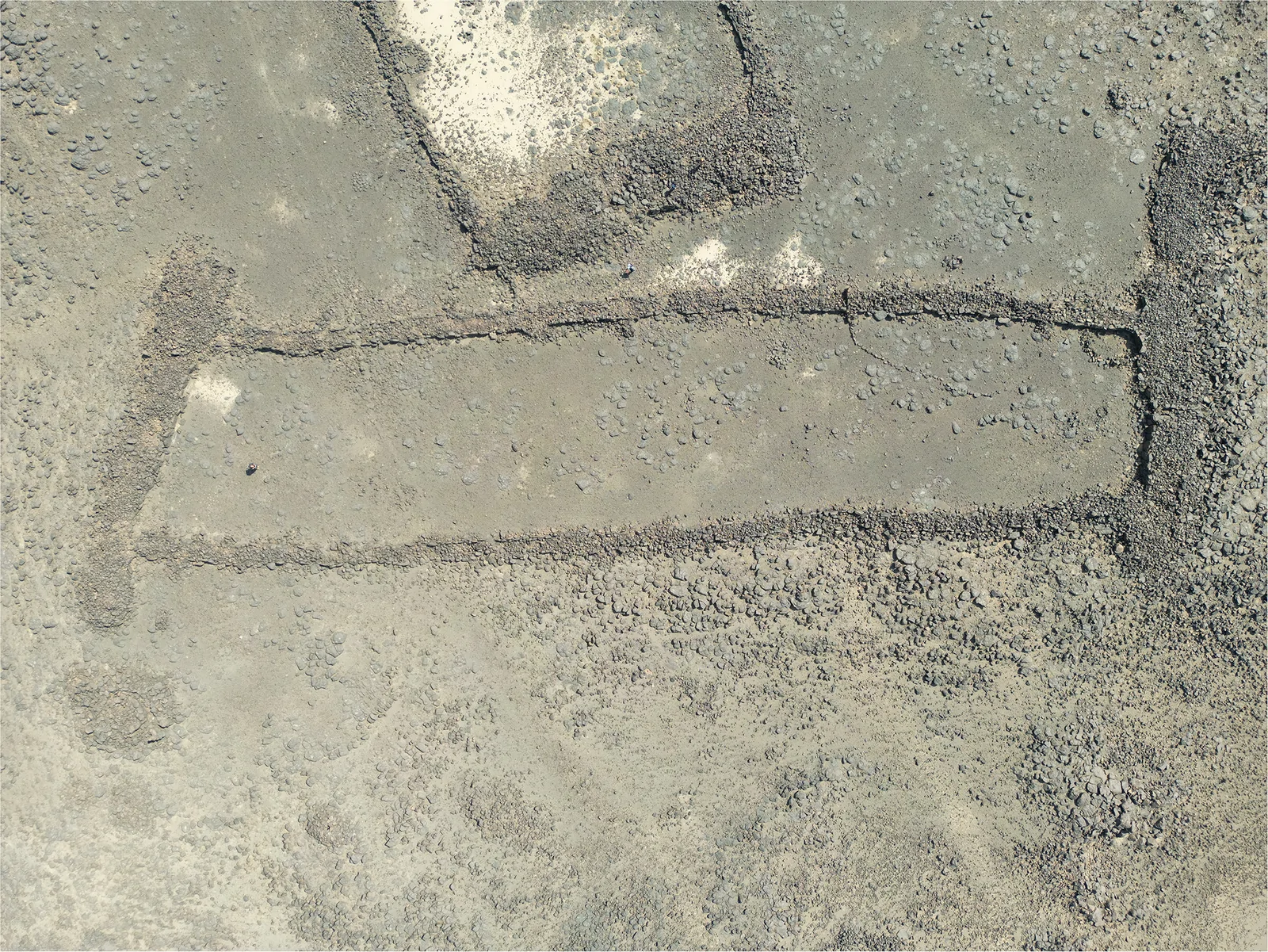
Aerial view of IDIHA-F-0051662 (Variant 1), photo orientated to the south-east. Base platform is approximately 30 m wide.

Variant 1 is the typical mustatil type outlined in Thomas *et al.* [3]. This is typified by IDIHA-F-0051662 (Fig 3), which was characterised by a sub-rectangular form with two sub-rectangular platforms, which form the head and base of the structure. The platforms are linked by two parallel long walls. There is a narrow entranceway through the base platform. Most of the mustatils at this site conform to this variant (n = 14).

### Variant 2

**Fig 4:**
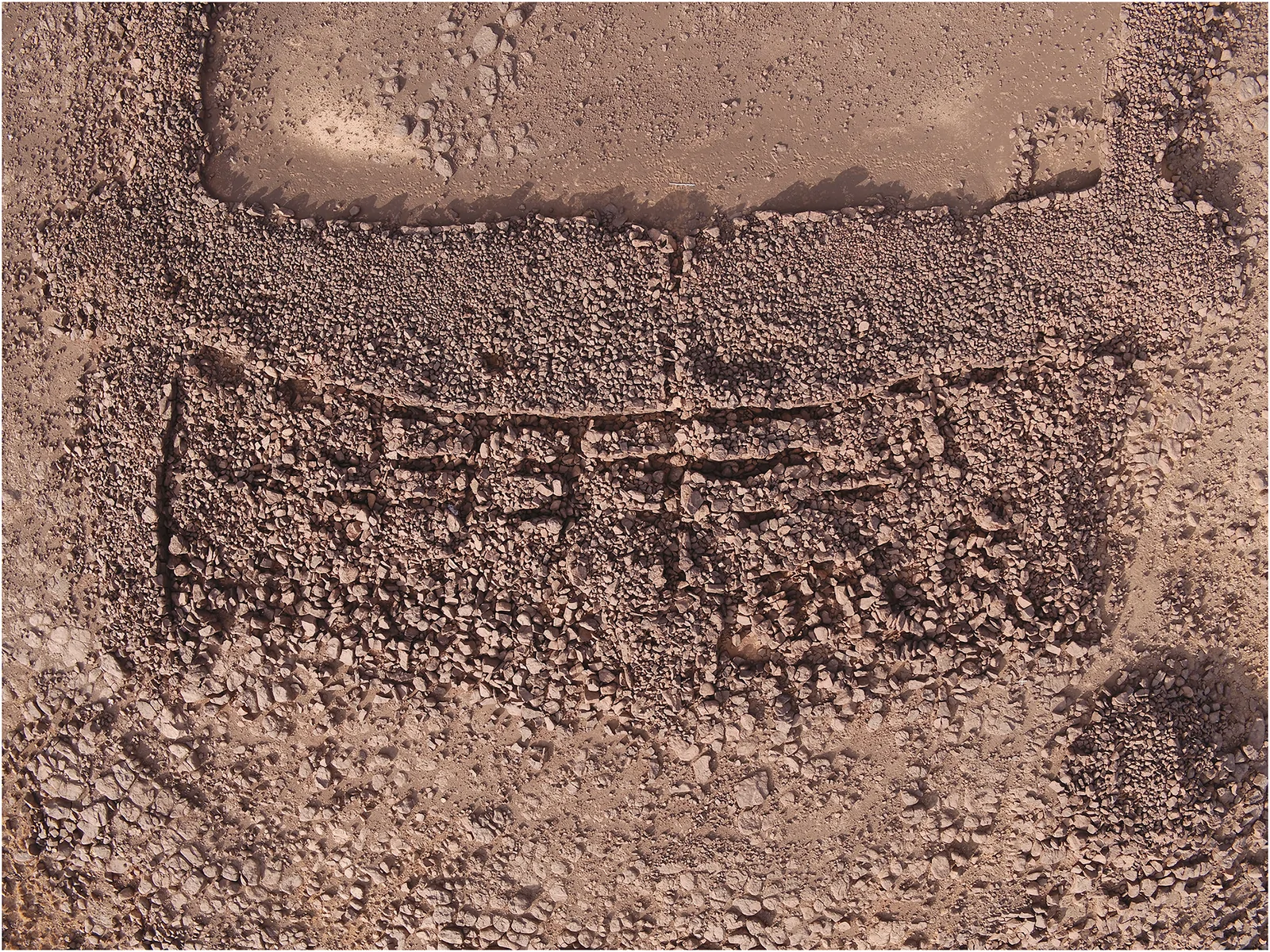
Aerial photograph of IDIHA-F-0050776 (Variant 2), note the tunnels in front of the base, photo taken to the east. Base platform is approximately 52 m wide.

**Fig 5:**
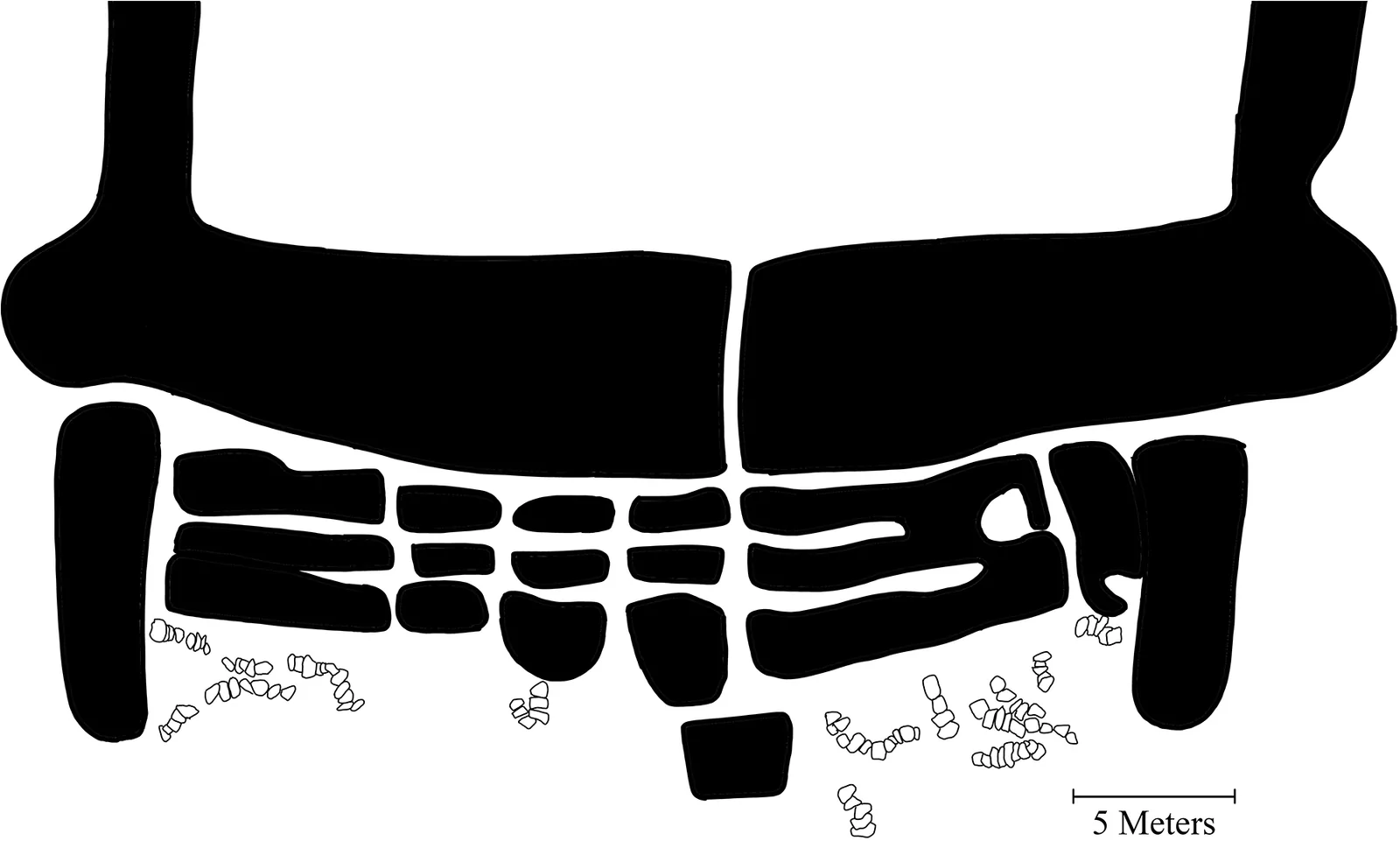
Schematic plan of the base of Variant 2 (IDIHA-F-0050776).

**Fig 6:**
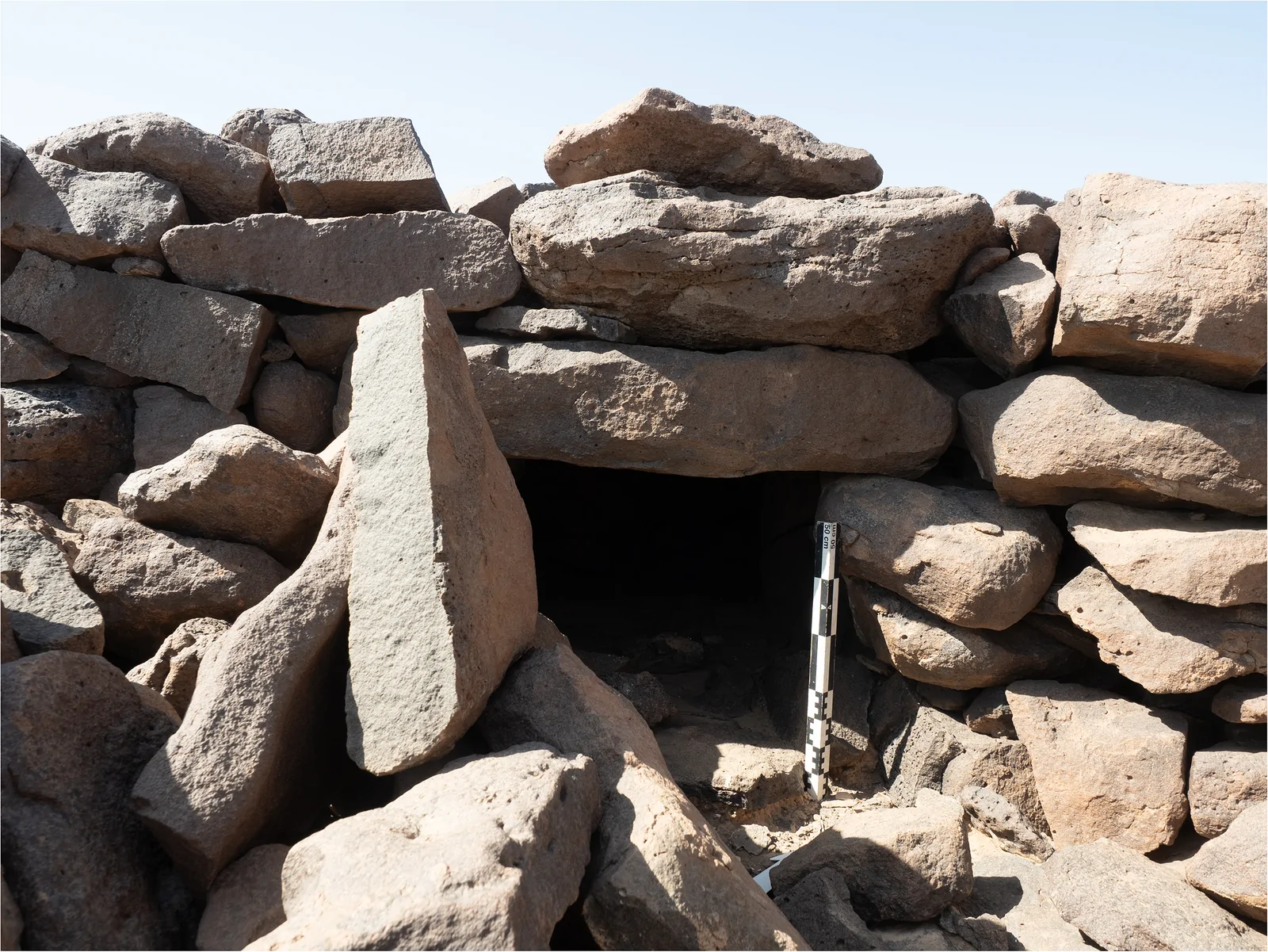
Detail of one of the tunnels at IDIHA-F-0050776.

**Fig 7:**
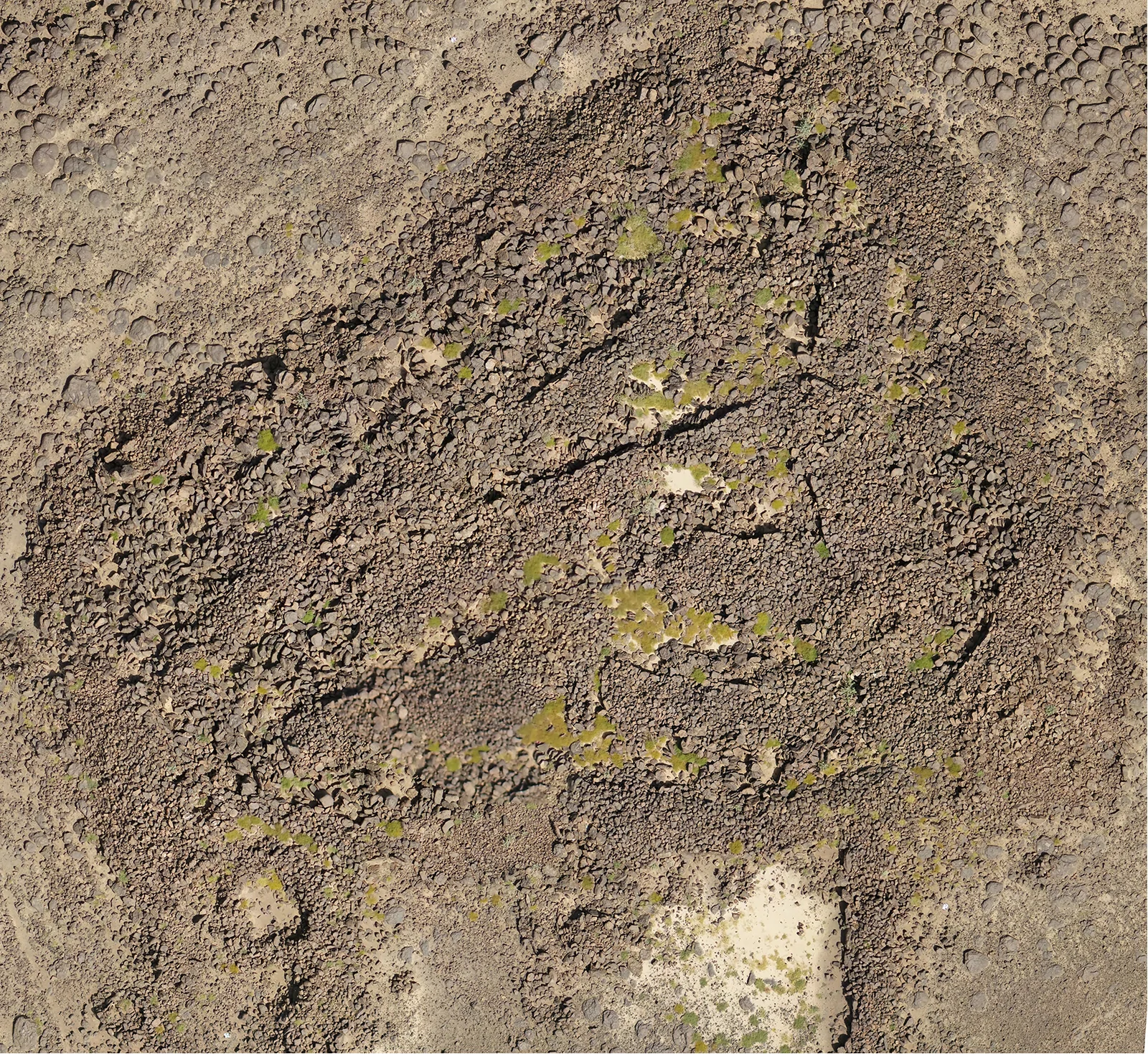
Overhead view of IDIHA-F-0011392, note the toppled pillars and maze-like structure surrounding the base of the structure, photo orientated to the north. Base platform is approximately 55 m wide.

Variant 2 is marked by a series of narrow tunnels, less than a metre high and wide, constructed in front of the base. Only a single example of Variant 2 was identified at the study site. IDIHA-F-0050776 is a complex mustatil orientated east-west, with the head in the east, measuring 160 m long and 51 m wide. The tunnels associated with this feature were formed by a line of nine roughly rectangular platforms measuring roughly 2 x 3 m, set in three parallel rows. Flat capstones, several of which were still *in situ*, were then set across the area between the long ends of each platform to form a tunnel (Figs 4 and 5). To enter the mustatil, individuals would have had to have crawled on their hands and knees through these tunnels to reach the entranceway in the base. There appears to be a central corridor/tunnel that runs directly from the exterior of the structure to the entranceway in the base, however several alternative routes can also be taken to access the entranceway. To create these tunnels, given the comparatively low height of the long walls and head, the architects used the underlying slope of the plateau to create more space/height, whilst the remainder of the structure, i.e., from the base onwards, was constructed on higher, relatively level ground. Additionally, rows of collapsed and *in situ* stone pillars were positioned directly in front of this platform/tunnel area. As with many of the mustatils of AlUla, the structure appears to have been decommissioned, with the tunnels and the entranceway blocked with stones and many of the stone pillars toppled (Fig 6). This is the only example of this type identified at this site, although another possible example is found approximately 23 km to the south, IDIHA-F-0011392. IDIHA-F-0011392 is one of the largest mustatils in the region, measuring 525 m in length. The base of this structure is enclosed by a sub-circular wall with a series of irregularly shaped, rock covered platforms, pathways, and rows upon rows of toppled stone pillars (Fig 7). However, this feature does not have the same overall structure height as IDIHA-F-0050776.

### Variant 3

**Fig 8:**
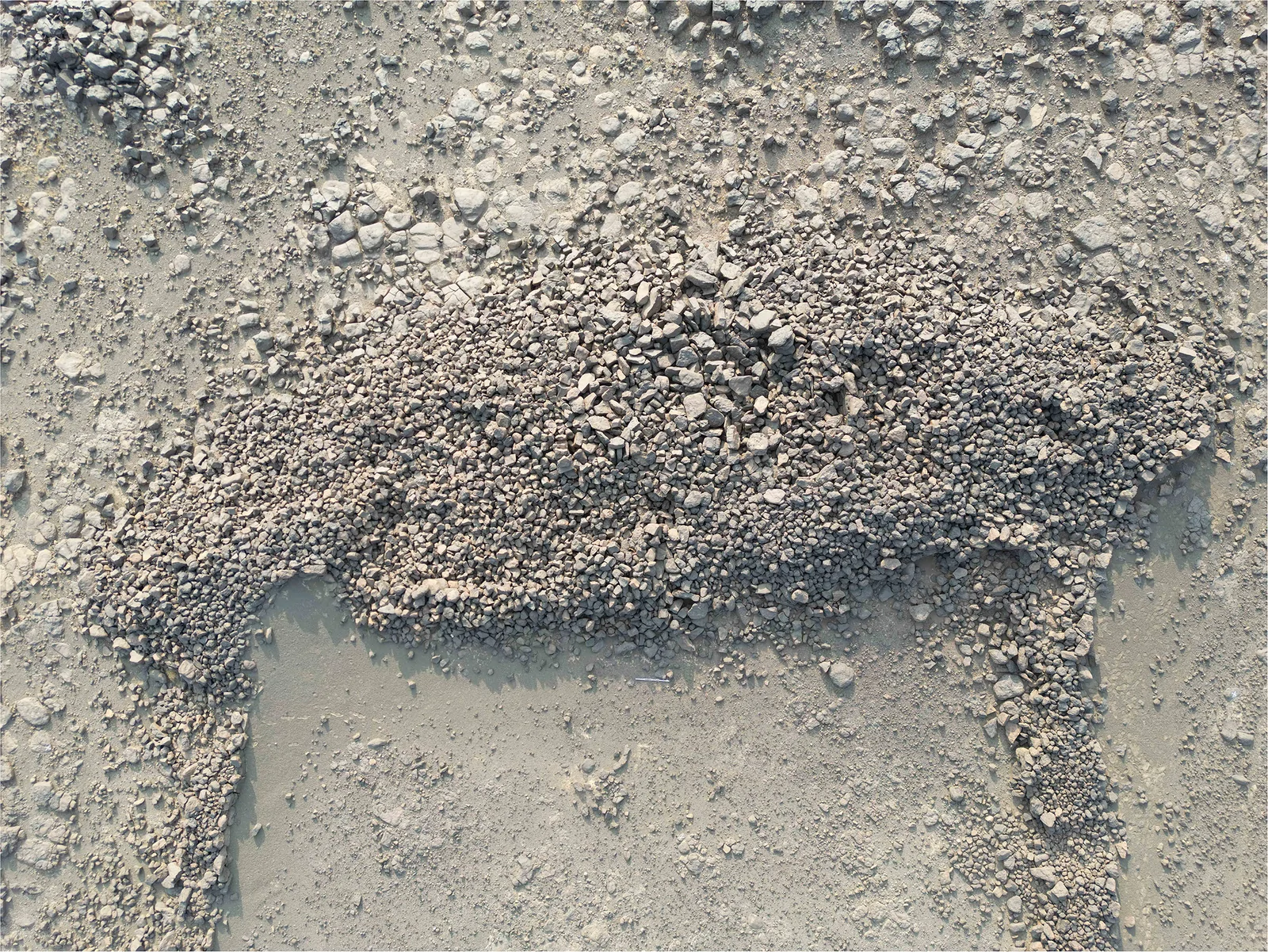
Aerial view of the base of IDIHA-F-0050739 (Variant 3), note the oval chamber outline. Base platform is approximately 32 m wide.

**Fig 9:**
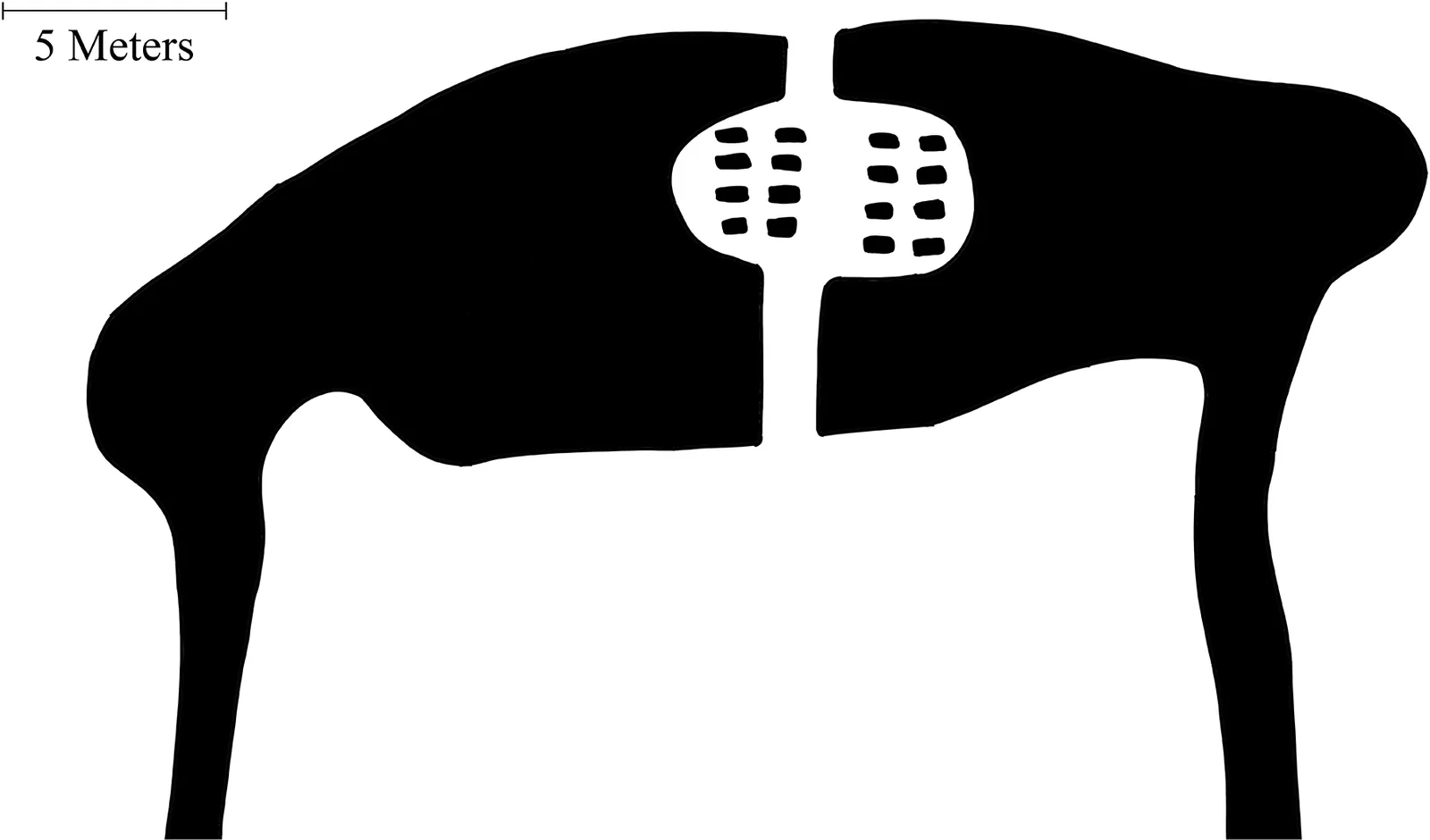
Schematic plan of the base form of Variant 3 (IDIHA-F-0050739).

**Fig 10:**
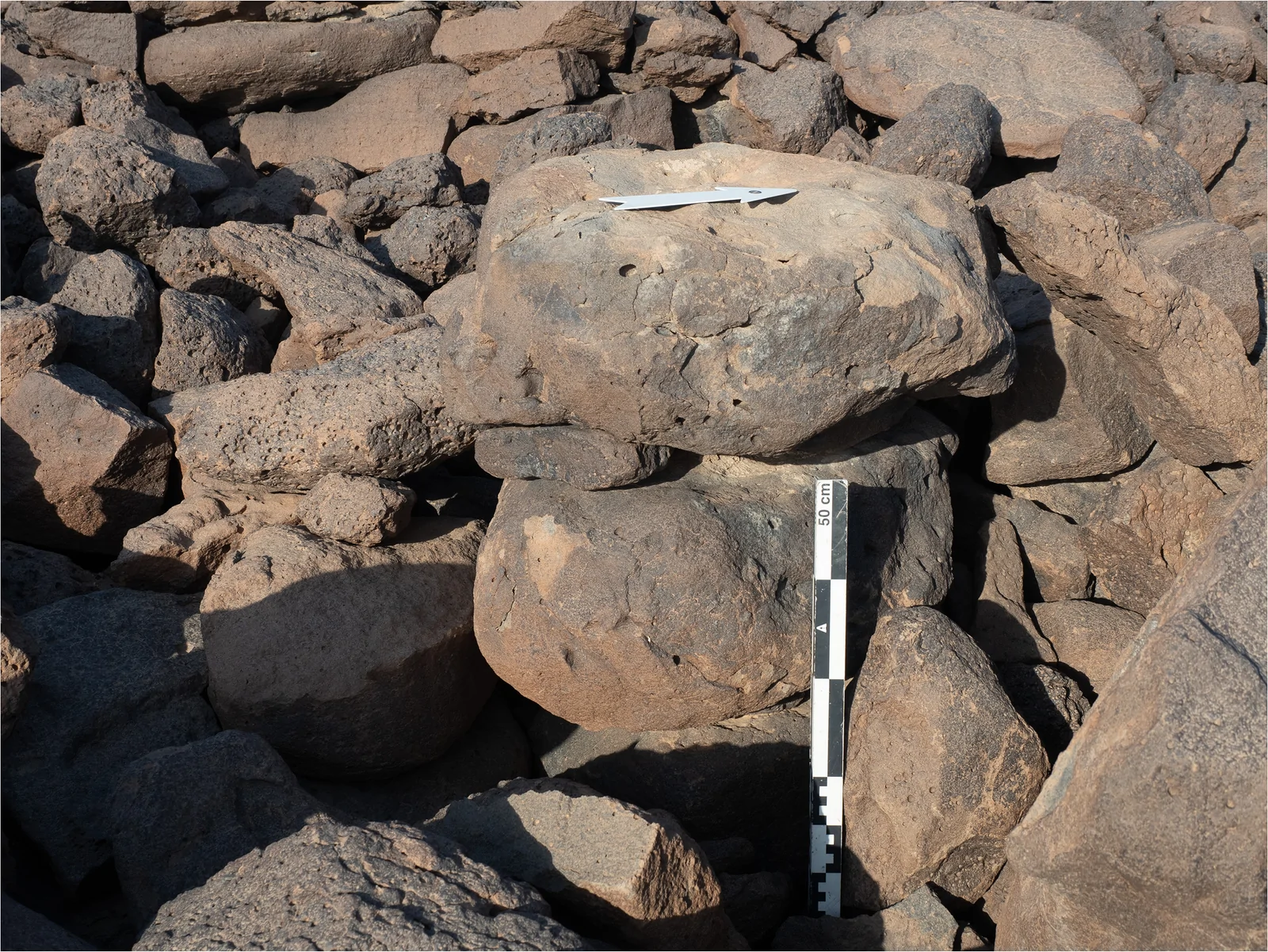
Pillars in the base chamber of IDIHA-F-0050796.

Variant 3 is marked by a circular or oval chamber in the base with a series of stone pillars set in rows (n = 3). This variant is exemplified by IDIHA-F-0050739, which lies 40 m to the south of IDIHA-F-0050776. This is a north-south orientated, sub-rectangular, complex mustatil. In contrast to the adjacent mustatils (IDIHA-F-0051631, IDIHA-F-0050065 and IDIHA-F-0050776), this structure was positioned parallel rather than perpendicular to the edge of the plateau. Measuring 138 m in length and 35 m in width (at its widest point), this feature was constructed from locally available basalt and was characterised by oval-shaped chambers in both the head and base. The base of this structure was approximately 32 m wide, within the centre of the platform was an approximately 11 m wide cell (Figs 8 and 9). In the eastern extent of the cell, at least two parallel rows of stone pillars were visible, which appear to have been extended in a similar configuration/pattern across the chamber. The full extent of this cell and the precise number of pillars could not be determined due to the large volume of stone collapse. A small space between these pillars was aligned with a 50–70 cm wide entranceway in the northern wall of the base, leading into the courtyard of the structure (Fig 10). A test excavation was conducted in this structure, the results of which will be discussed below. Several mustatils at this site were marked by this base variant, including IDIHA-F-0050796 and IDIHA-F-005079

### Variant 4

**Fig 11:**
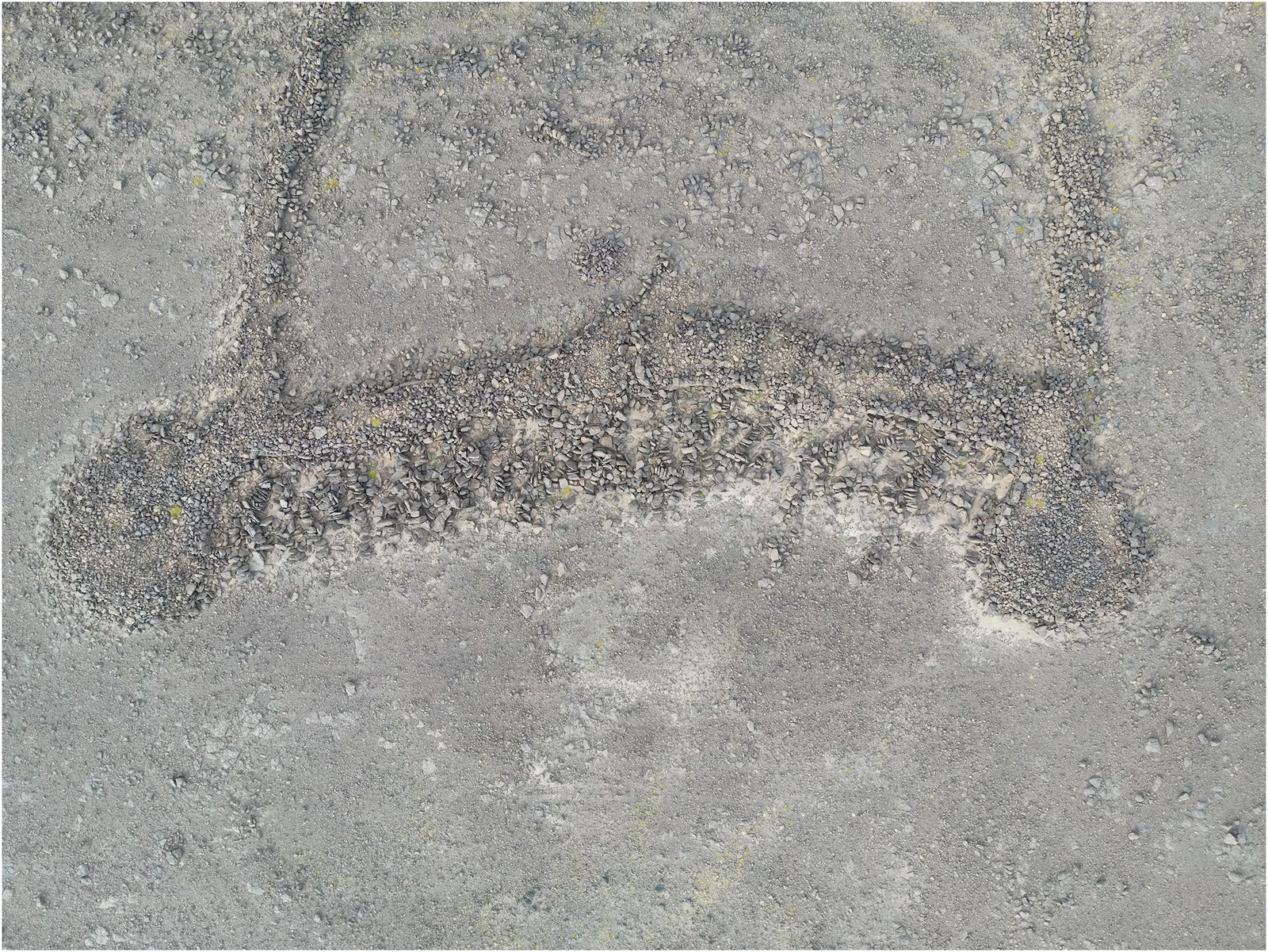
Aerial view of IDIHA- F-0051636, photo orientated to the north-east. Base platform is approximately 63 m wide.

**Fig 12:**
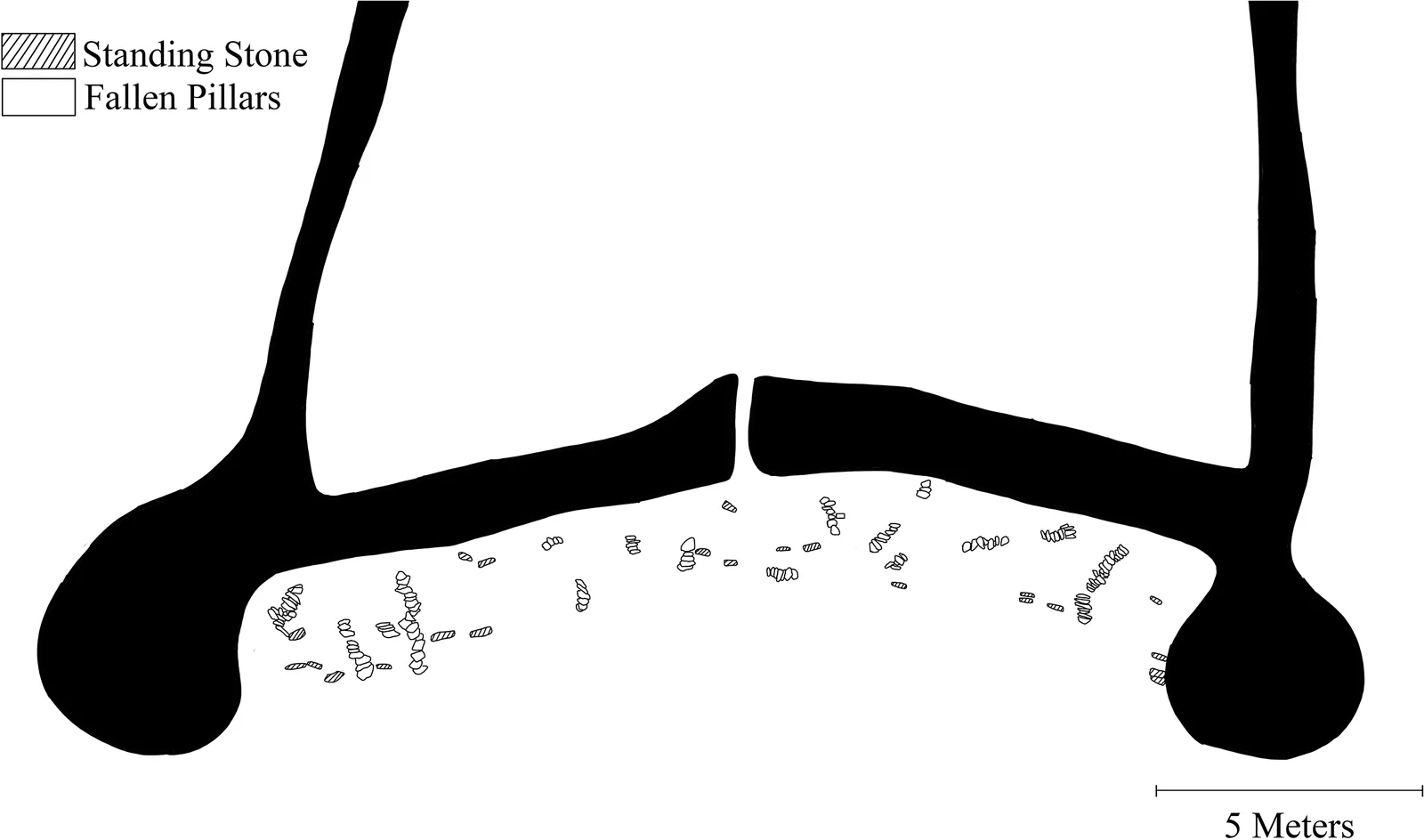
Schematic plan of the base of Variant 4 (IDIHA-F-0051636).

**Fig 13:**
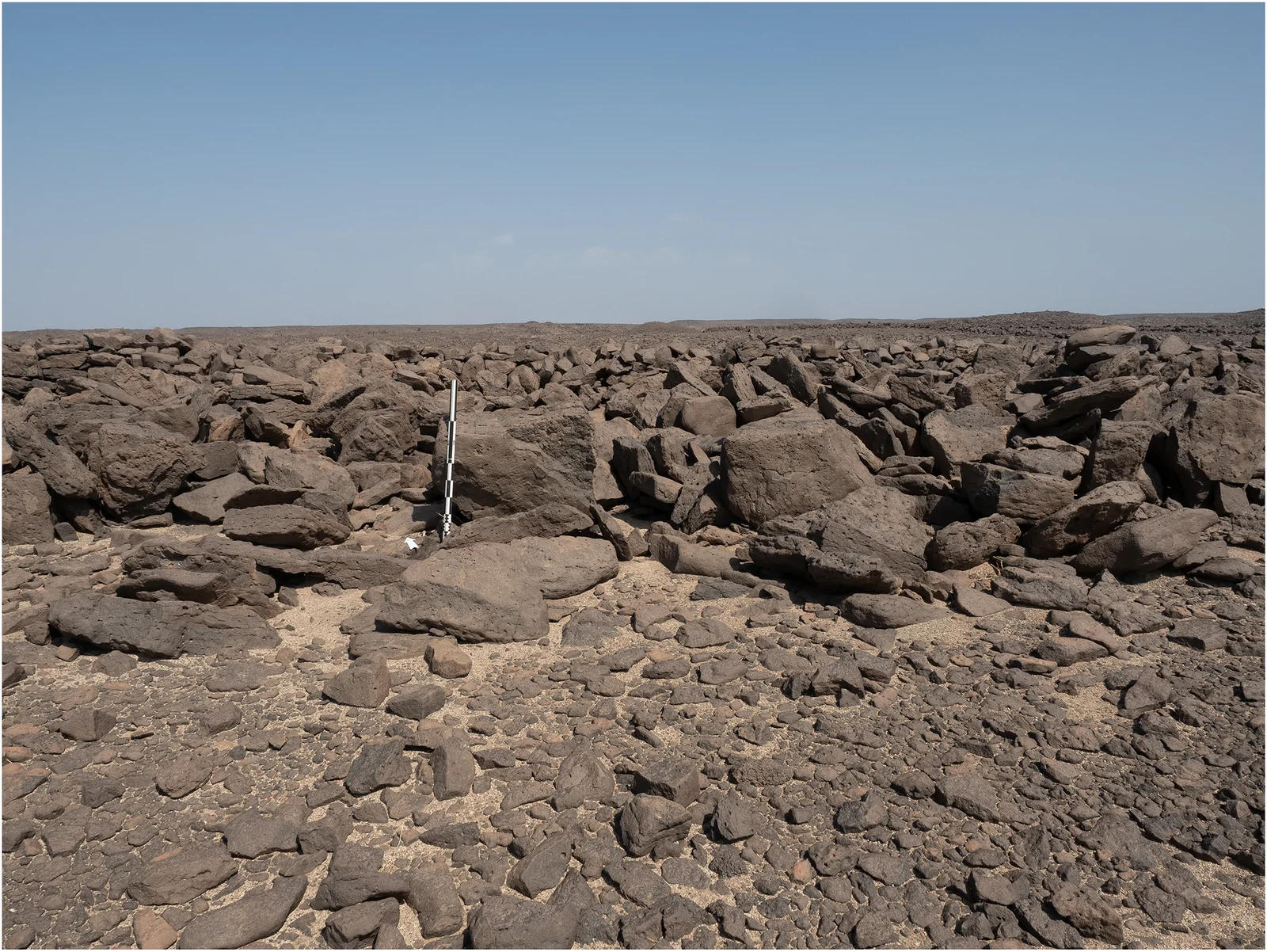
Orthostats and pillars associated with the base of IDIHA-F-0051636.

Variant 4 is marked by its crescent shaped base (n = 3). One of the best examples of this variant is IDIHA-F-0051636. This northeast-southwest running mustatil measured approximately 191 m by 47 m (at its widest point). The overall form of this structure and variant is sub-rectangular, with the rounded ends of the base protruding away from the courtyard, past the base platform, creating a crescent shape (Figs 11 and 12). Running in alignment between these two ends was a row of large basalt orthostats, with 22 orthostats recorded at this mustatil (Fig 13). These orthostats appear to delineate the beginning of the structure and its sacred space. Some of these stones were as high as 75 cm, weighing several hundred kilograms. Inside this orthostat line were a series of collapsed pillars (Figure 13). The pillars were composed of between 7–10 stones and were often more than 1.4 m high, which was taller than the mustatil. The courtyard of the structure was accessed by between one to three entranceways, determining the precise number of entranceways was difficult due to rubble infilling. The presence of multiple entranceways into a single mustatil is rare, and potentially an architectural feature limited to the Harrat Khaybar. It is of interest that the shape of the chamber from IDIHA-F-0051636 mirrored the form of the base. This apparent mirroring is present across several of mustatils from the study area. However, whether this is a deliberate design feature remains unknown.

### Excavation results

Test excavations were conducted in three mustatils: IDIHA-F-0050739, IDIHA-F-0051633 and IDIHA-F-0050796. The result of this work is as follows.

### IDIHA-F-0050739

**Fig 14:**
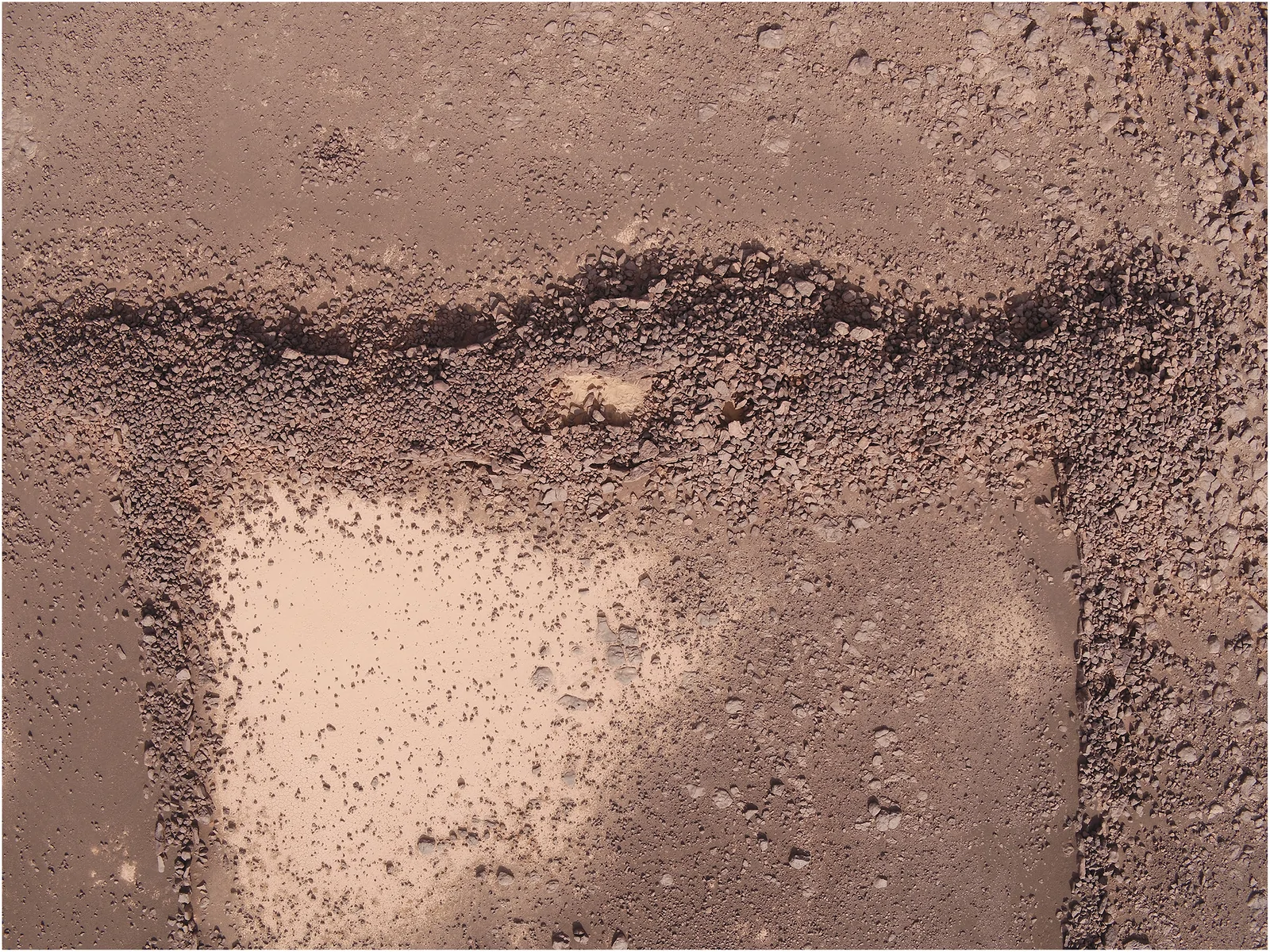
The head of IDIHA-F-0050739 (Variant 3) prior to excavation, note the clearly visible chamber, photo orientated to the north-west. Head platform is approximately 47 m wide.

**Fig 15:**
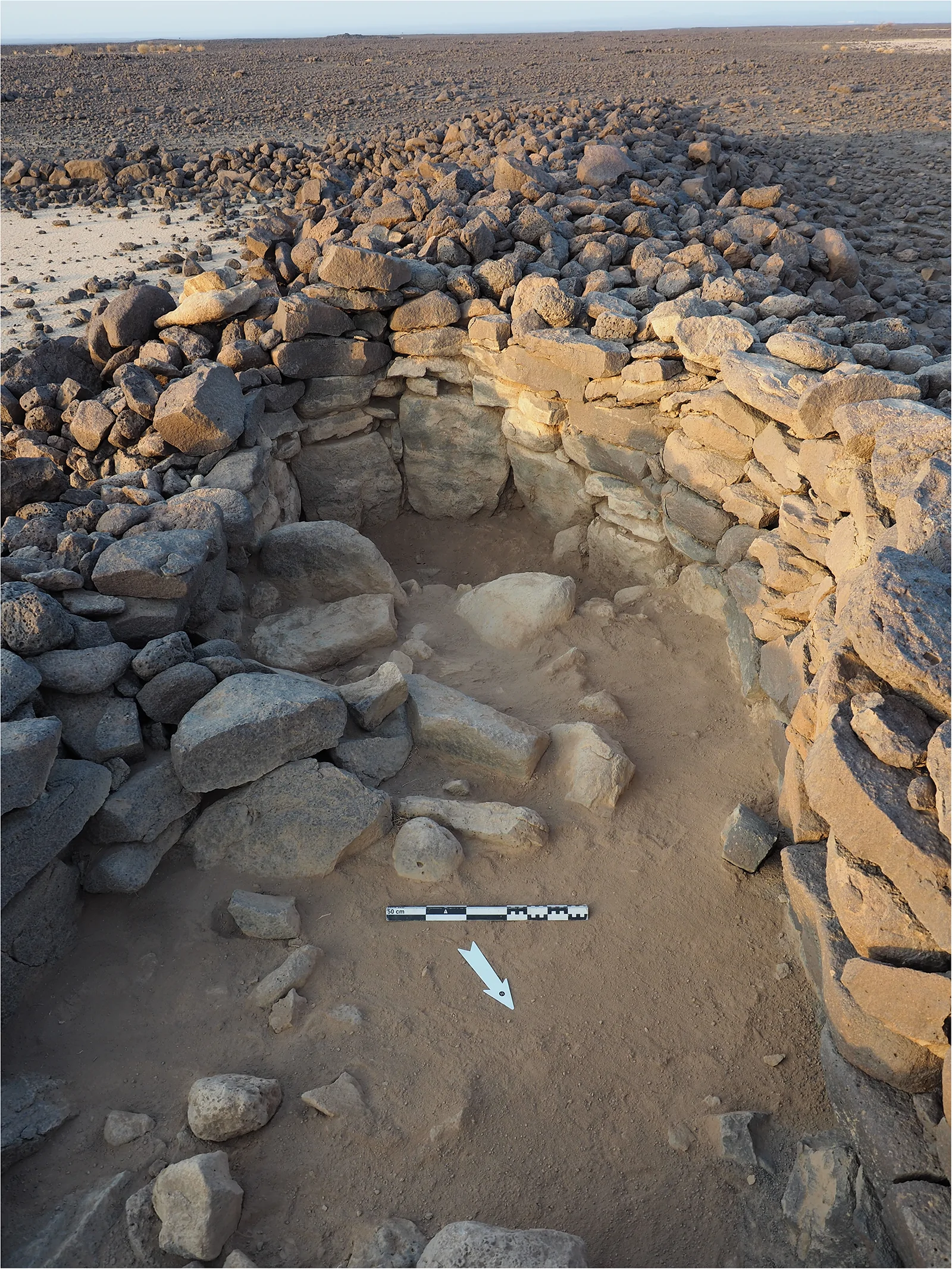
The chamber of IDIHA-F-0050739 (Variant 3) during excavation.

**Fig 16:**
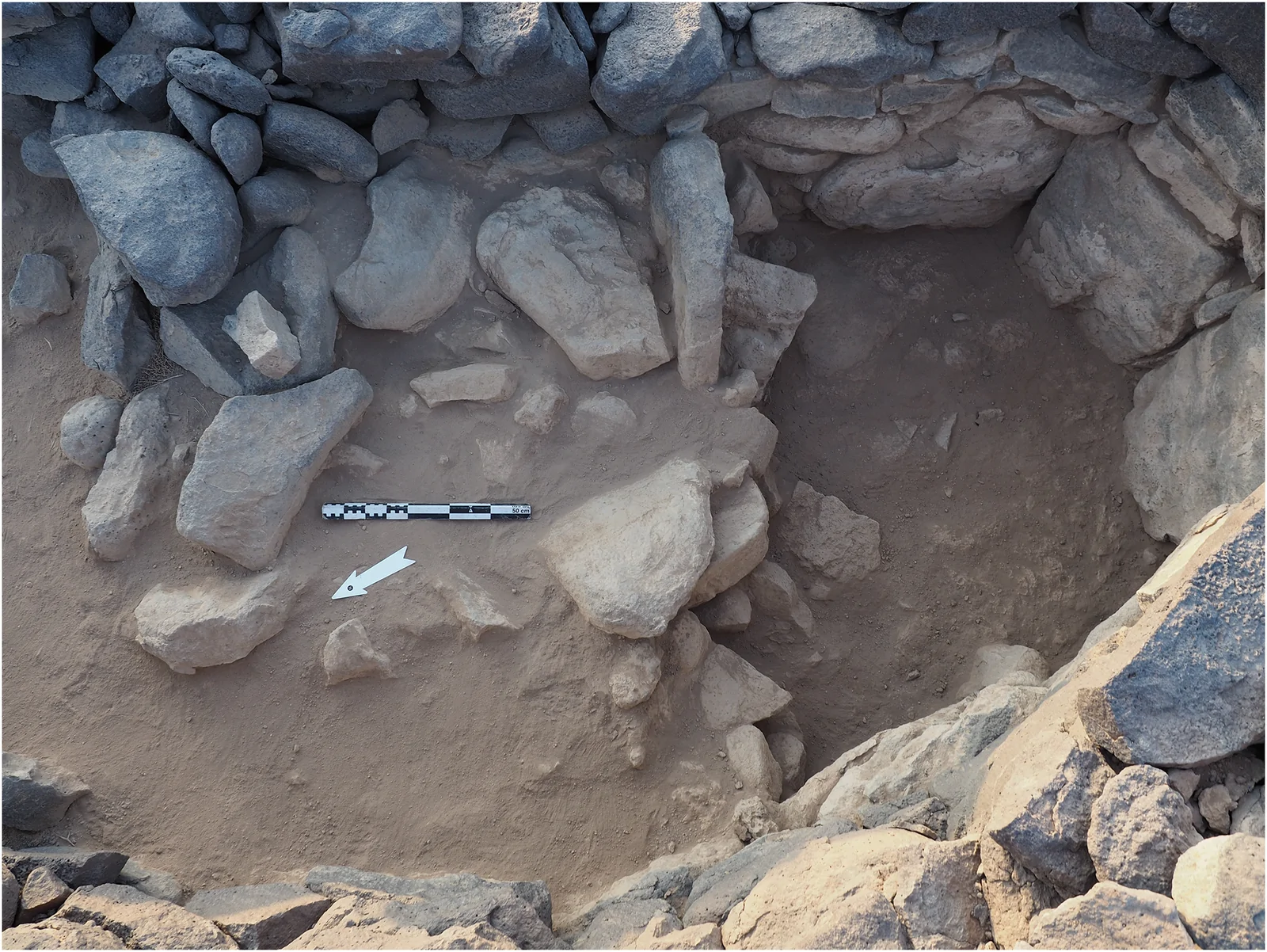
The chamber of IDIHA-F-0050739 (Variant 3) during excavation.

IDIHA-F-0050739 (Variant 3 – see above for detailed description of this mustatil) was chosen for excavation as an oval chamber was clearly visible in the head, with significant deposition present (Fig 14). A sondage measuring 1.8 x 2.2 m was excavated in the western extent of the chamber (Figs 15 and 16). Excavations revealed a single phase of use represented by seven stratigraphic contexts. The first stratigraphic unit, Context (001) consisted of basalt rubble which ranged in depth from 5 to 65 cm. This context sealed a 10−20 cm deposit of light brown, sandy-silty fill with sub-angular basalt stones (002). Within (002) was an installation of horizontally and vertically placed stone slabs (003), measuring 1.58 x 1.27 m. The precise function of this installation is unknown, although it does not appear to have been structural and was too large to be removed, it may in fact represent a capping layer. As this installation was left *in situ*, excavation shifted to the south, with the area reduced to 1.4 x 1.25 m. Context (004) was an arbitrary change from (002). Animal tooth enamel flakes and bone fragments were found throughout (004), as was evidence for bioturbation, with insect casings and snake eggshells present throughout matrix. Due to the apparent disturbance of this context (004) no samples were radiocarbon dated from this deposit. Context (004) sealed (005), a context of horizontally laid stone which extended across the excavation area. These stones may have functioned as a partial sealing layer for the underlying deposits. Beneath (005) was (006), a layer of loose, soft, silty grey fill rich in faunal remains. Several cattle petrous bones and numerous cattle and *Bos* sp. tooth fragments were found throughout (006). This horizon appears to mark the primary depositional event of this mustatil, with the finds from contexts (001) to (005) most probably secondary in nature, the result of bioturbation and other unknown taphonomic processes. Of note, no clear floor level was identified, with the faunal elements placed directly on the natural soil profile (007), directly on bedrock (008). A single sample from (006) was sent for radiocarbon dating (bioapatite). This sample had a 2σ date spread of 4796−4620 cal. BC, with a median date of 4739 cal. BC. No other finds were recovered from this structure.

**Fig 17:**
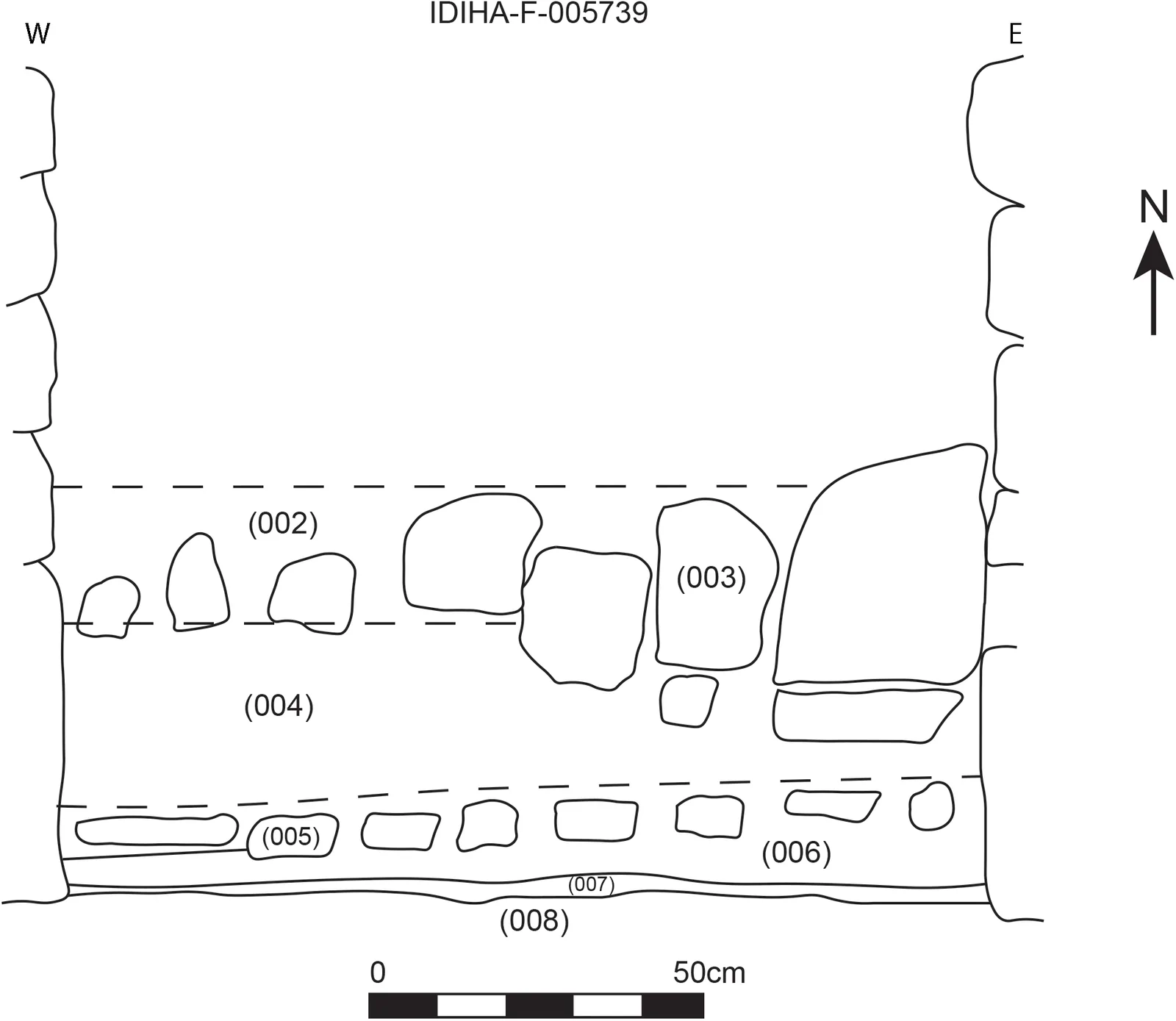
North section of excavation area in IDIHA-F-0050739.

As only a small portion of the chamber was excavated only a preliminary summary can be offered (Fig 17). Based on our interpretation of the excavated sequence we would assert that a single faunal deposit was present within IDIHA-F-0050739 (006). This deposit showed no later anthropic interventions, with the stone installation (003) possibly related to the final use of the structure, potentially as a capping/sealing. However, the chamber was characterised by significant bioturbation (002 & 004), which resulted in some faunal remains found above the primary deposit of (006).

### IDIHA-F-0051633

**Fig 18:**
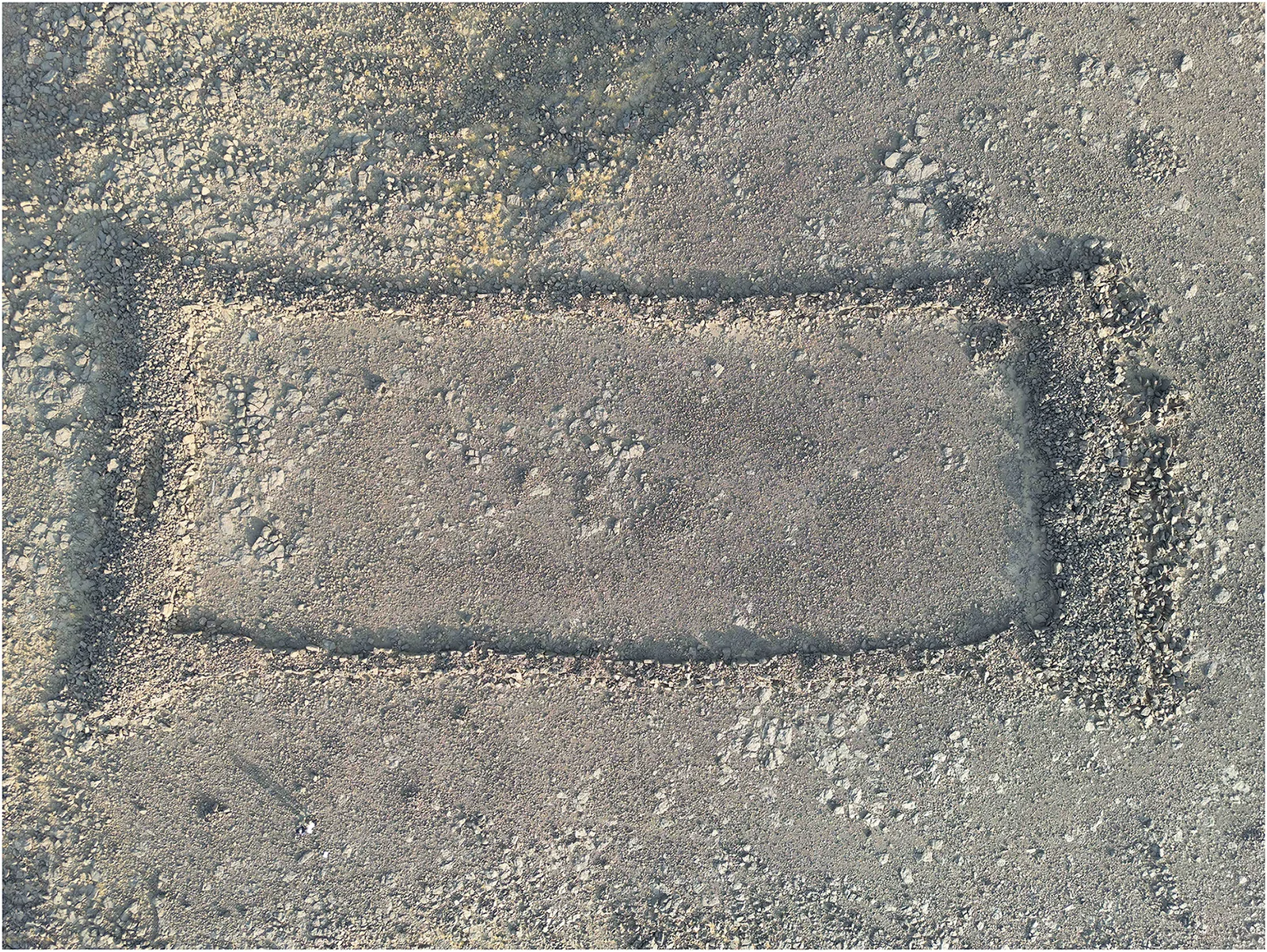
Pre-excavation aerial photograph of IDIHA-F-0051633 (Variant 1), photo orientated to the north. Note the visible chamber in the head platform in the east (left of photo). Head platform measures approximately 32 m in width, the sounding was executed in the southern extent of the chamber.

**Fig 19:**
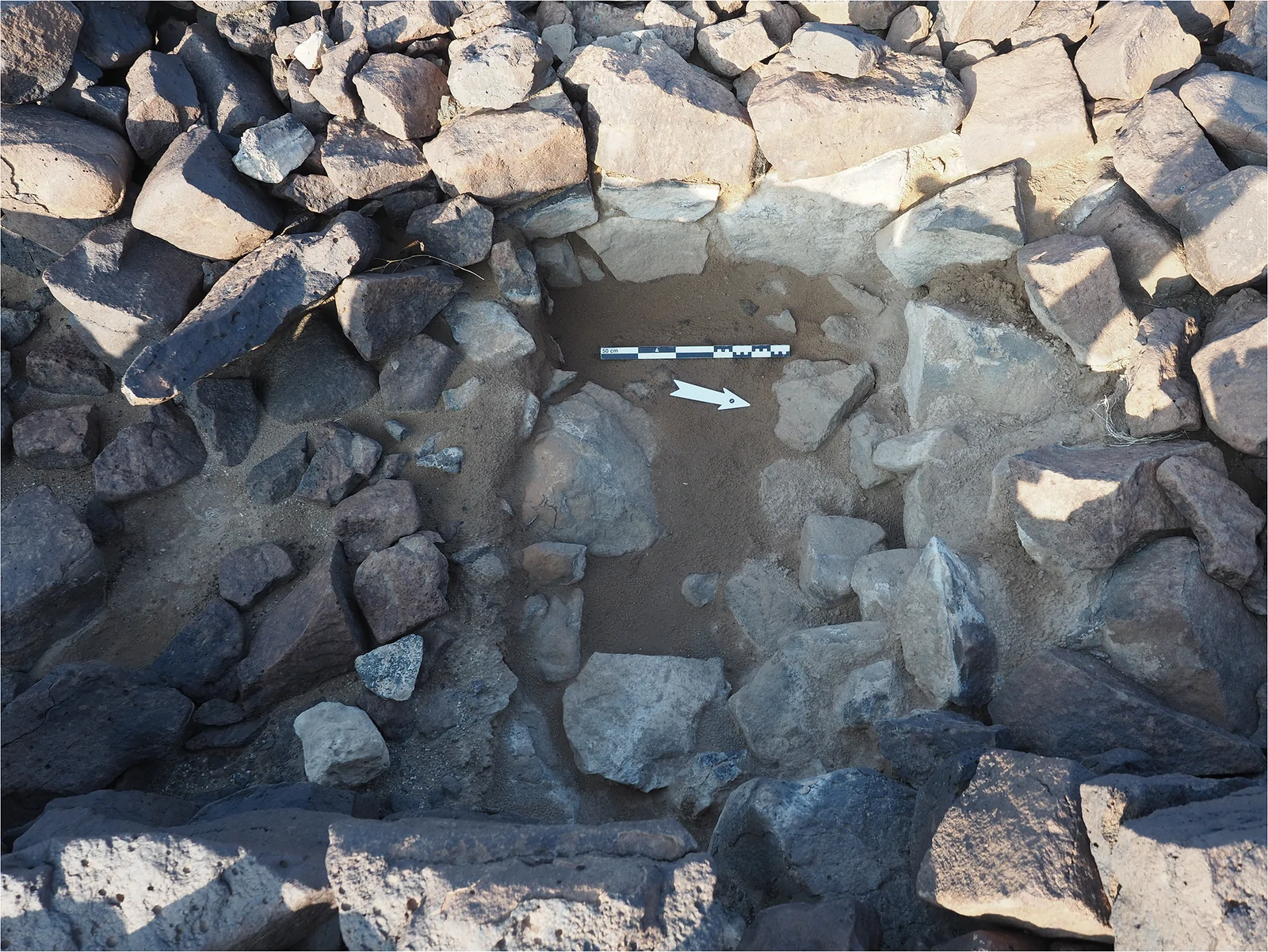
Sondage 1 in IDIHA-F-0051633 (Variant 1).

**Fig 20:**
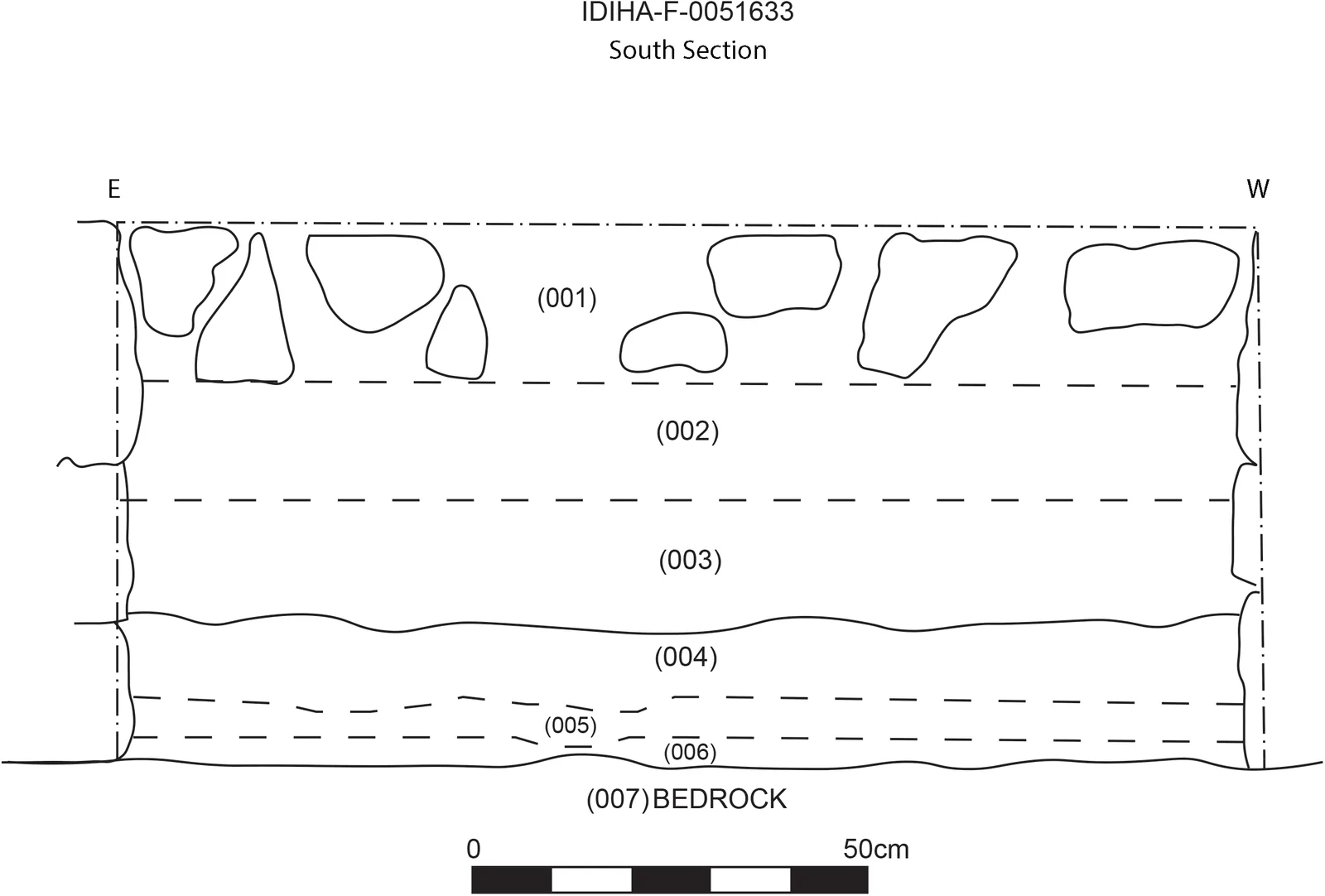
South Section of excavation area in IDIHA-F-0051633.

IDIHA-F-0051633 is an east-west orientated, complex mustatil (Variant 1), constructed from locally available, unworked basalt boulders. This structure measured 69 x 32 m, with the head located in the west, and the chamber clearly visible (Fig 18). At least one clear entranceway was visible in the base, with a further two possible entranceways also noted. Ten standing orthostats were also present here, with a series of collapsed pillars positioned between these stones and the base platform. A 1 x 1.45 m sondage was executed in the northern part of the chamber (Fig 19), with excavations revealing two distinct phases of use (based on radiocarbon data) across six contexts. Context (001) consisted of basalt collapse/rubble. Beneath (001) were Contexts (002) and (003), a 15 cm deposit of loose grey silty-sandy fill with gravel and stone inclusions. Context (003) was an arbitrary change, with little stratigraphic differentiation evident. Animal bone fragments and tooth enamel flakes were found throughout these two contexts. Beneath (003) was (004) a mottled brown silty-sand. Faunal enamel tooth and bone flakes increased in frequency throughout this this context. Following rain, an arbitrary change was made from (004) to (005), to avoid potential contamination. This deposit was also characterised by a medium brown silty-sandy fill. Once the silty-sand fill was removed, the natural soil profile (006) and bedrock (007) was identified across the excavation area. Two radiocarbon dates were obtained from this mustatil, the first from (004) had a 2σ date spread of 4537−4365 cal. BC, with a median probability of 4458 cal. BC. The second date was gleaned from the earlier (lower) Context (005) had a 2σ spread of 4696−4539 cal. BC, with a median probability of 4611 cal. BC (Fig 20).

Based on the preliminary excavation data and the radiometric assays presented above, IDIHA-F-0051633 appears to have had two distinct phases of animal offerings. These two phases were not stratigraphically discernible during excavation, most probably due to environmental factors, such as long-term exposure to the elements and bioturbation/taphanomic processes (Fig 20).

### IDIHA-F-0050796

**Fig 21:**
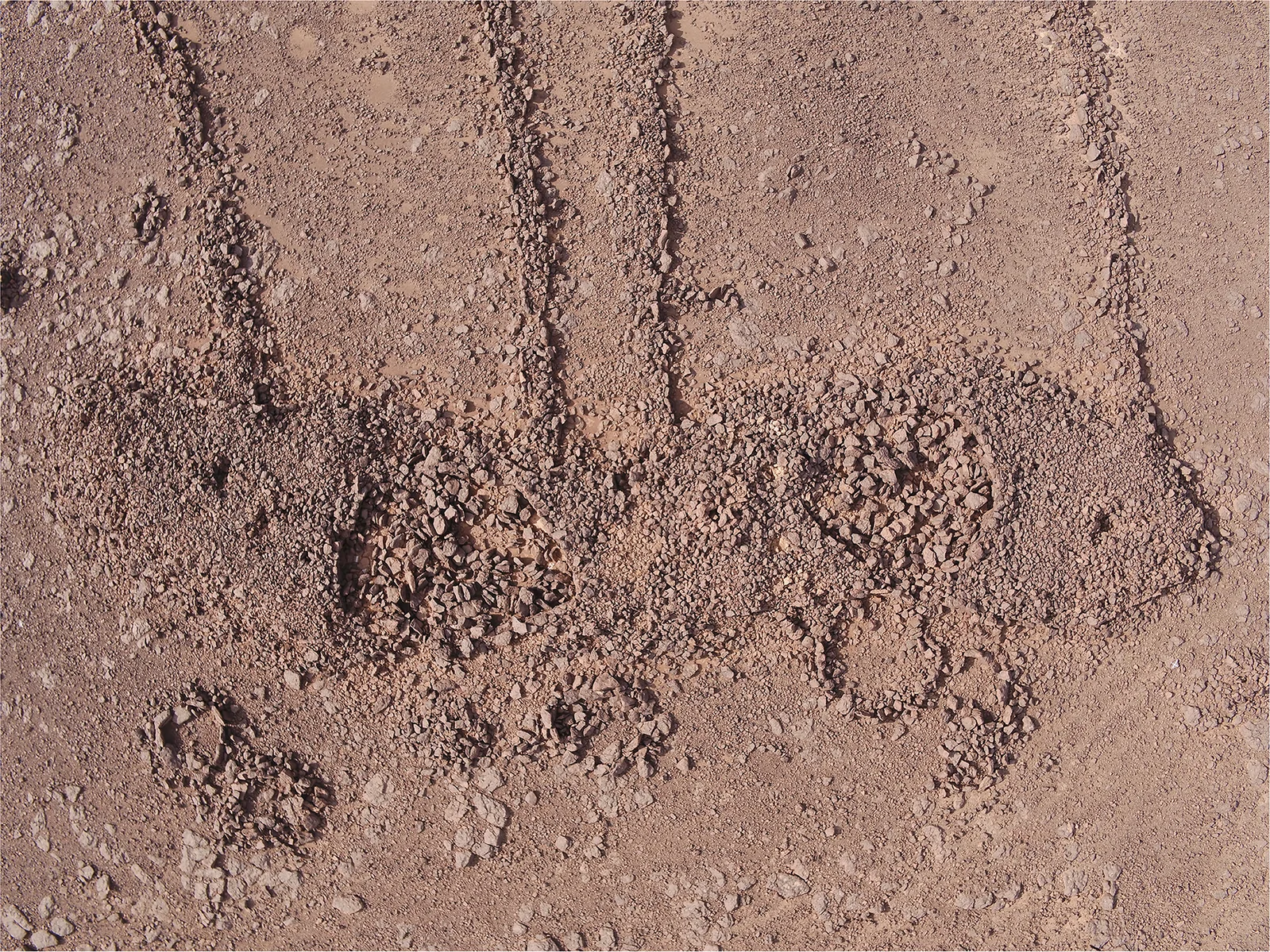
Aerial view of the bases of IDIHA-F-0050796 (left) and IDIHA-F-0050797 (right) – Variant 3, photo orientated to the south-west. Each platform is approximately 25 m in width.

**Fig 22:**
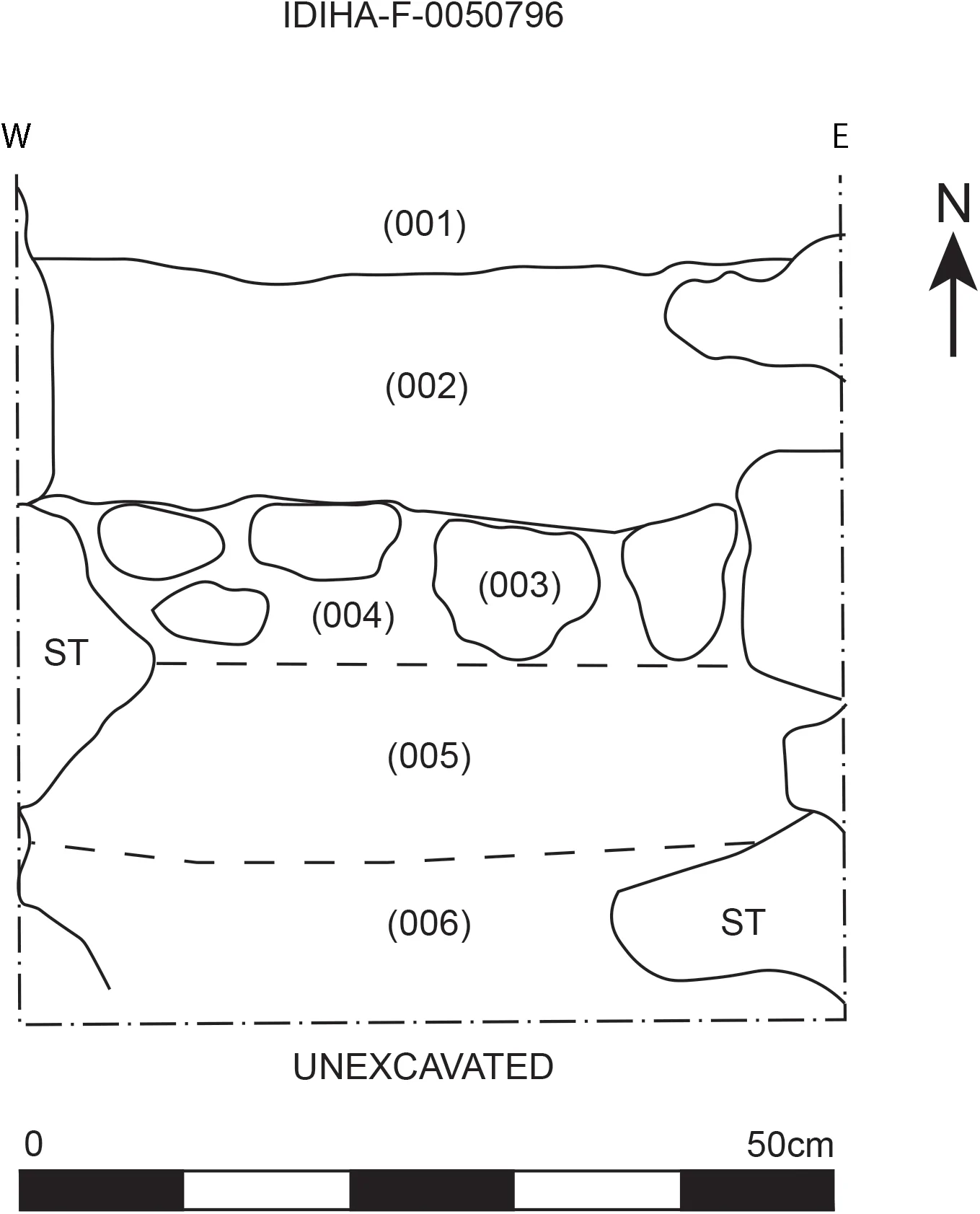
North Section of excavation area in IDIHA-F-0050796.

A third sondage was conducted in IDIHA-F-0050796 (Variant 3). This mustatil measured 290 x 32 m and was orientated roughly northeast-southwest, with the head in the southwest. Interestingly, this structure is one of two identical mustatils, built side by side (IDIHA-F-0050797). Both mustatils were characterised by oval cells with pillars in the base, identical to IDIHA-F-0050739 (Variant 3; Fig 21). Due to time constraints only a small 0.5 x 0.5 m sondage was undertaken in the western extent of the chamber against the rear wall (Figure 21). Based on the excavated data, only a single phase of use, represented by five contexts could be discerned in this structure. The limited scope of these results is due to the fact that excavations ceased prior to the identification of bedrock (Fig 22). The first context (001) consisted of loose, silty-sand with gravel. This context sealed (002), a deposit of medium brown silty-sand overlaying a 15−20 cm layer of basalt stones (003). Beneath (003) was (004), a 5 cm deep context of light brown silty-sand, a single animal tooth enamel flake was recovered from this context. Context (005) was an arbitrary change and was compositionally identical to (004). The final context, (006), consisted of a compact brown silty-sandy matrix with white mottling. Animal bone and tooth enamel flakes were recovered from the northwest and southwest sections of the sondage. A radiocarbon date of 4709−4547 cal. BC (2σ), with a median probability of 4635 BC was obtained from this feature. Due to the small size of the excavation, little interpretative framework from this structure can be proposed. No other finds were recovered from this structure.

### Sampled mustatils

**Fig 23:**
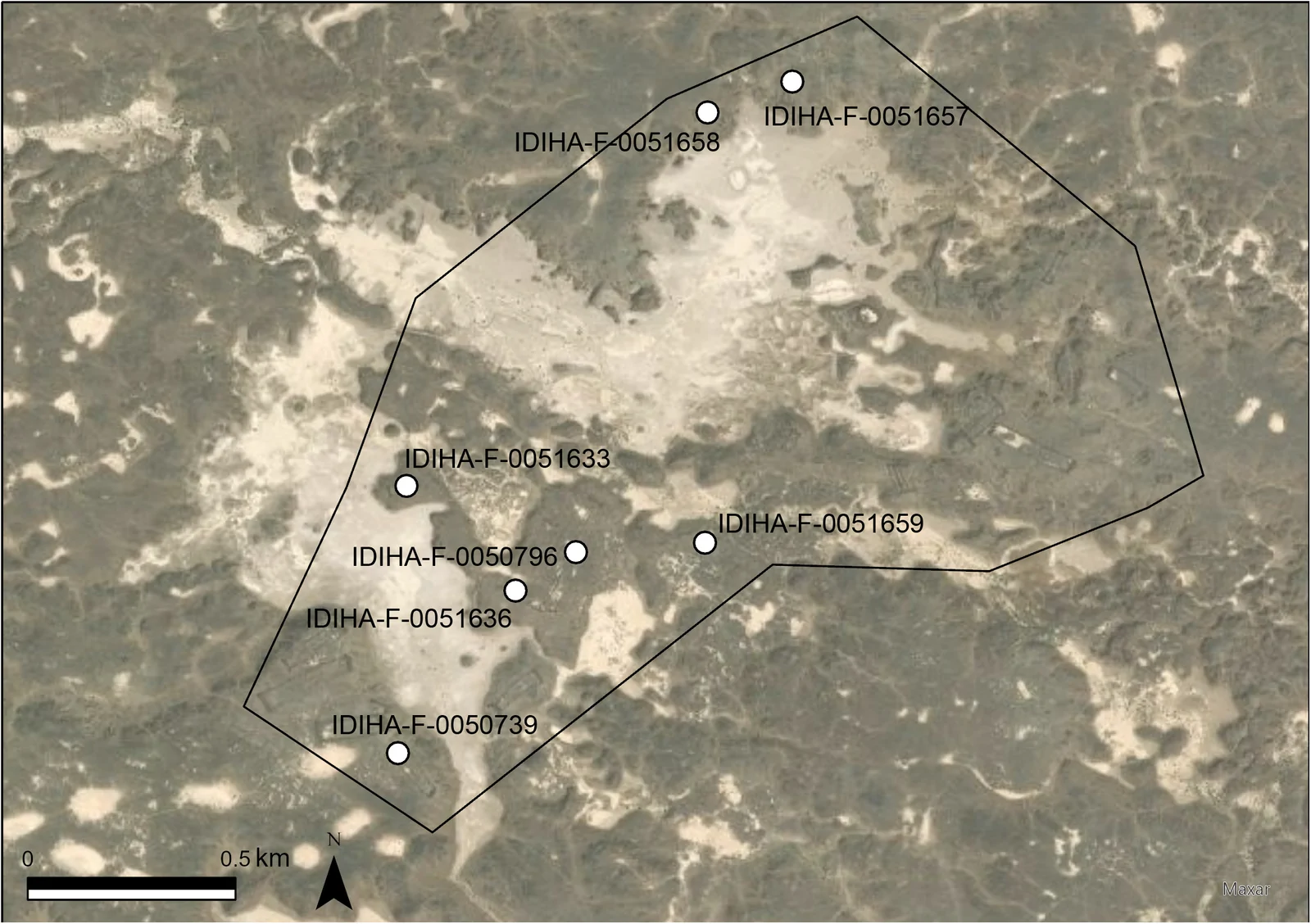
Excavated and sampled mustatils from the study site. Basemap source: Esri, Maxar, Earthstar Geographics, and the GIS User Community.

**Fig 24:**
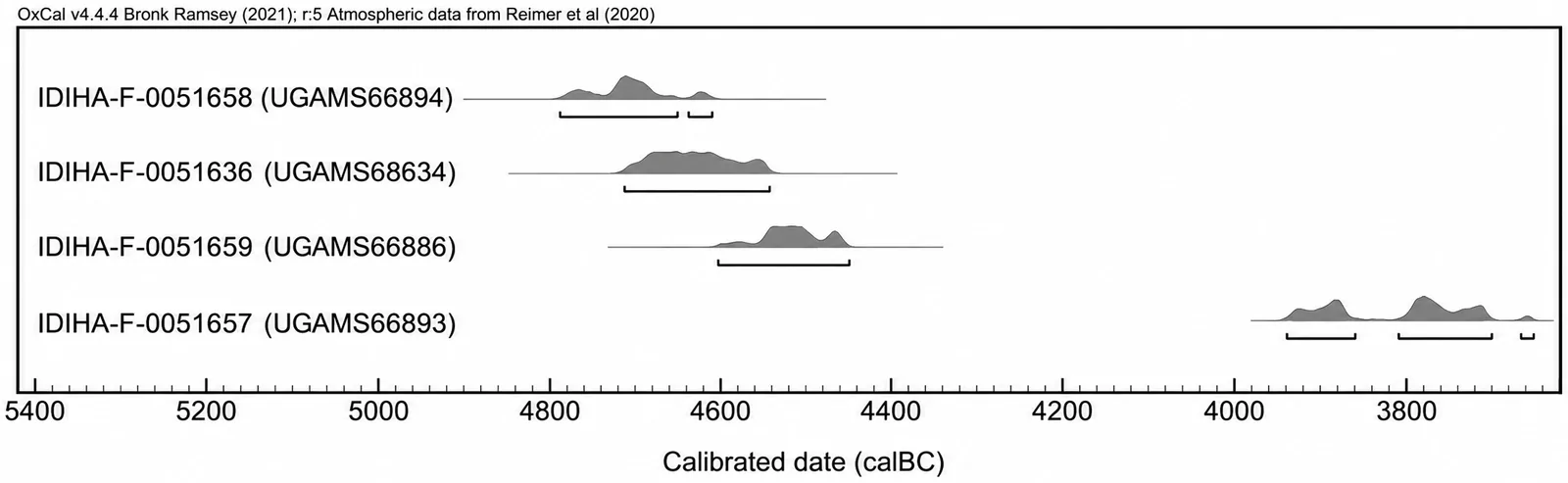
Radiocarbon assays from surface sampled mustatils at Al-Thamad.

In addition to test excavations, all 28 mustatils at the site were ground surveyed. Based on our previous work in AlUla, we have determined that it is possible to obtain datable surface material associated with the use of the mustatils (Fig 23). Using surface material for dating purposes can be problematic. When considering using surface material issues such as recovery bias and a lack of stratigraphic control are potential factors that need to be considered with their use. However, in some instances, the use of surface materials is the only way to obtain datable material from a mustatil. Not all mustatils contain intact depositional sequences; many have been looted to bedrock or have suffered from significant structural robbing, removing most, if not all relevant *in situ* archaeological material. To avoid dating contaminated or unrelated materials, a strict collection policy was undertaken: only bone and enamel tooth fragments were collected from the chamber of the mustatil (with 100% of visible faunal material collected), with only cattle enamel tooth fragments radiocarbon dated (with the exception of UGAMS66893, see below). Given the high concentration of cattle within the mustatils excavated, and the fact that these animals are no longer herded at scale across the Saudi Arabia, the likelihood that these remains are intrusive is low [6]. To date, we have excavated two mustatils which were initially dated by surface samples (cattle tooth enamel fragments), these dates were in good accordance with those recovered during full-scale excavation [6]. Although this methodology does not provide a comprehensive overview of the original assemblage and phasing/use of the structure, it does provide a non-invasive and time sensitive way of gaining a small chronological insight in to at least one phase of use. Based on this approach and with these caveats in mind we were able to obtain datable material from the following mustatils listed in Fig 24 and Table 1.

**Table 1:** Radiocarbon assays from surface sampled mustatils at Al-Thamad.

Feature	Lab ID	Sample ID	Material	Analysis	14C (BP)	Calibrated Date BC 68.2% (1σ)	Calibrated Date BC 95.4% (2σ)
IDIHA-F-0051658	UGAMS66894	#1817	Cattle Tooth	Bioapatite	5840 ± 25	4779−4753 (0.223) 4728−4680 (0.770) 4627−4625 (0.007)	4788−4653 (0.910) 4638−4614 (0.090)
IDIHA-F-0051636	UGAMS68634	#1754	Cattle Tooth	Bioapatite	5780 ± 30	4689−4598 (0.0923) 4564−4555 (0.077)	4709−4547 (1.000)
IDIHA-F-0051659	UGAMS66886	#1821	Cattle Tooth	Bioapatite	5690 ± 25	4544−4492 (0.859) 4472−4460 (0.141)	4602−4562 (0.101) 4556−4453 (0.899)
IDIHA-F-0051657	UGAMS66893	#1820	Cattle Tooth	Bioapatite	5010 ± 25	3907−3878 (0.287) 3802−3760 (0.481) 3741−3712 (0.232)	3943−3863 (0.353) 3812−3706 (0.630) 3670−3660 (0.017)

### Summary of faunal remains from the mustatils

Faunal remains were recovered from ten mustatils at the Al-Thamad site, through a combination of both excavation and ground survey. In total, 681 faunal remains (347.8 g) were collected from the study area, with the quantification per single feature shown in Table 2. A total of 638 animal bones (327.4 g) were recovered from the excavated structures (IDIHA-F0050739, IDIHA-F0051633 and IDIHA-F-0050796). As for the other seven mustatils, IDIHA-F-0051636, IDIHA-F-0051657, IDIHA-F-0051658, IDIHA-F-0051659, IDIHA-F-0051664, IDIHA-F-0051665 and IDIHA-F-0051666, a total of 43 (21.4 g) animal bones were recovered. Despite the relatively small size of the assemblage, these results shed new light on and give important insight into the mustatils of Khaybar County, as well as our understanding of the broader mustatil phenomenon [4–6].

**Table 2:** Quantification of faunal remains from the mustatils of the study area at Al-Thamad. The faunal remains from excavated mustatils (IDIHA-F-0050739; IDIHA-F-0051633 and IDIHA-F-0050796) are listed first, followed by the faunal remains from surface sampling (indicated by *; IDIHA-F-0051636; IDIHA-F-0051657; IDIHA-F-0051658; IDIHA-F-0051659; IDIHA-F-0051664 IDIHA-F-0051665; IDIHA-F-0051666).

IDIHA-F-0050739				
Taxa	NR	% NR	weight (g)	% weight
Cattle	259	50.5%	190.8	70.6%
*Bos* sp.	2	0.4%	1	0.4%
Indeterminate Caprine	1	0.2%	1	0.4%
Small Ruminant	2	0.4%	0.3	0.1%
Ruminant	36	7.0%	10.3	3.8%
Large Ungulate	2	0.4%	2.4	0.9%
Rodent	8	1.6%	0.8	0.3%
Indeterminate	203	39.6%	63.6	23.5%
**Total**	**513**	**100.0%**	**270.2**	**100.0%**
**IDIHA-F-0051633**				
**Taxa**	**NR**	**% NR**	**weight (g)**	**% weight**
Cattle	43	36.4%	21.8	39.4%
Indeterminate Caprine	3	2.5%	1.1	2.0%
Small Ruminant	10	8.5%	2.7	4.9%
Ruminant	2	1.7%	0.9	1.6%
Large Ungulate	2	1.7%	4	7.2%
Hare	1	0.8%	0.8	1.4%
Indeterminate	57	48.3%	24	43.4%
**Total**	**118**	**100.0%**	**54.4**	**100.0%**
**IDIHA-F-0050796**				
**Taxa**	**NR**	**% NR**	**weight (g)**	**% weight**
Cattle	3	42.9%	1.2	63.2%
Indeterminate	4	57.1%	0.7	36.8%
**Total**	**7**	**100.0%**	**1.9**	**100.0%**
IDIHA-F-0051636*				
**Taxa**	**NR**	**% NR**	**weight (g)**	**% weight**
*Bos* sp.	2	13.3%	1.5	27.3%
Indeterminate	13	86.7%	4	72.7%
**Total**	**15**	**100.0%**	**5.5**	**100.0%**
IDIHA-F-0051657*				
**Taxa**	**NR**	**% NR**	**weight (g)**	**% weight**
Large Ungulate	1	6.3%	1	11.4%
Small Ruminant	1	6.3%	2	22.7%
Indeterminate	14	87.5%	5.8	65.9%
**Total**	**16**	**100.0%**	**8.8**	**100.0%**
IDIHA-F-0051658*				
**Taxa**	**NR**	**% NR**	**weight (g)**	**% weight**
Cattle	2	40.0%	2	62.5%
Large Ungulate	2	40.0%	0.5	15.6%
Indeterminate	1	20.0%	0.7	21.9%
**Total**	**5**	**100.0%**	**3.2**	**100.0%**
IDIHA-F-0051659*				
**Taxa**	**NR**	**% NR**	**weight (g)**	**% weight**
Cattle	1	100.0%	1	100.0%
**Total**	**1**	**100.0%**	**1**	**100.0%**
IDIHA-F-0051664*				
**Taxa**	**NR**	**% NR**	**weight (g)**	**% weight**
Cattle	3	100.0%	0.7	100.0%
**Total**	**3**	**100.0%**	**0.7**	**100.0%**
IDIHA-F-0051665*				
**Taxa**	**NR**	**% NR**	**weight (g)**	**% weight**
Small Ruminant	1	50.0%	1	50.0%
Indeterminate	1	50.0%	1	50.0%
**Total**	**2**	**100.0%**	**2**	**100,0%**
IDIHA-F-0051666*				
**Taxa**	**NR**	**% NR**	**weight (g)**	**% weight**
Small Ruminant	1	100.0%	0.2	100.0%
**Total**	**1**	**100.0%**	**0.2**	**100.0%**
**Faunal remains from mustatil surface sampling (7 mustatils)**				
**Taxa**	**NR**	**% NR**	**weight (g)**	**% weight**
Cattle	6	14.0%	3.7	17.3%
*Bos* sp.	2	1.5%	1.5	7.0%
Small Ruminant	3	7.0%	3.2	15.0%
Large Ungulate	3	7.0%	1.5	7.0%
Indeterminate	29	11.5%	11.5	53.7%
**Total**	**43**	**100.0%**	**21.4**	**100.0%**
**Combined total of all mustatils (excavated and surface sampled)**				
**Taxa**	**NR**	**% NR**	**weight (g)**	**% weight**
Cattle	311	45.7%	217.5	62.4%
*Bos* sp.	4	0.6%	2.5	0.7%
Indeterminate Caprine	4	0.6%	2.1	0.6%
Small Ruminant	15	2.2%	6.2	1.8%
Ruminant	38	5.6%	11.2	3.2%
Large Ungulate	7	1.0%	7.9	2.3%
Hare	1	0.1%	0.8	0.2%
Rodent	8	1.2%	0.8	0.2%
Indeterminate	293	43.0%	99.8	28.6%
**Total**	**681**	**100.0%**	**347.8**	**100.0%**

### Excavated faunal remains

A total of 638 faunal remains were retrieved from the three excavated mustatils, as detailed in Table 2. Out of this total, 264 fragments (41.4%) could not be identified to species level, due to poor preservation. Indeed, faunal remains from all the mustatils were found in a sub-optimal state of preservation. Notably, 391 of the remains consist of tooth fragments, with enamel flakes predominating (270, 69% of the teeth).

### IDIHA-F-0050739

**Fig 25:**
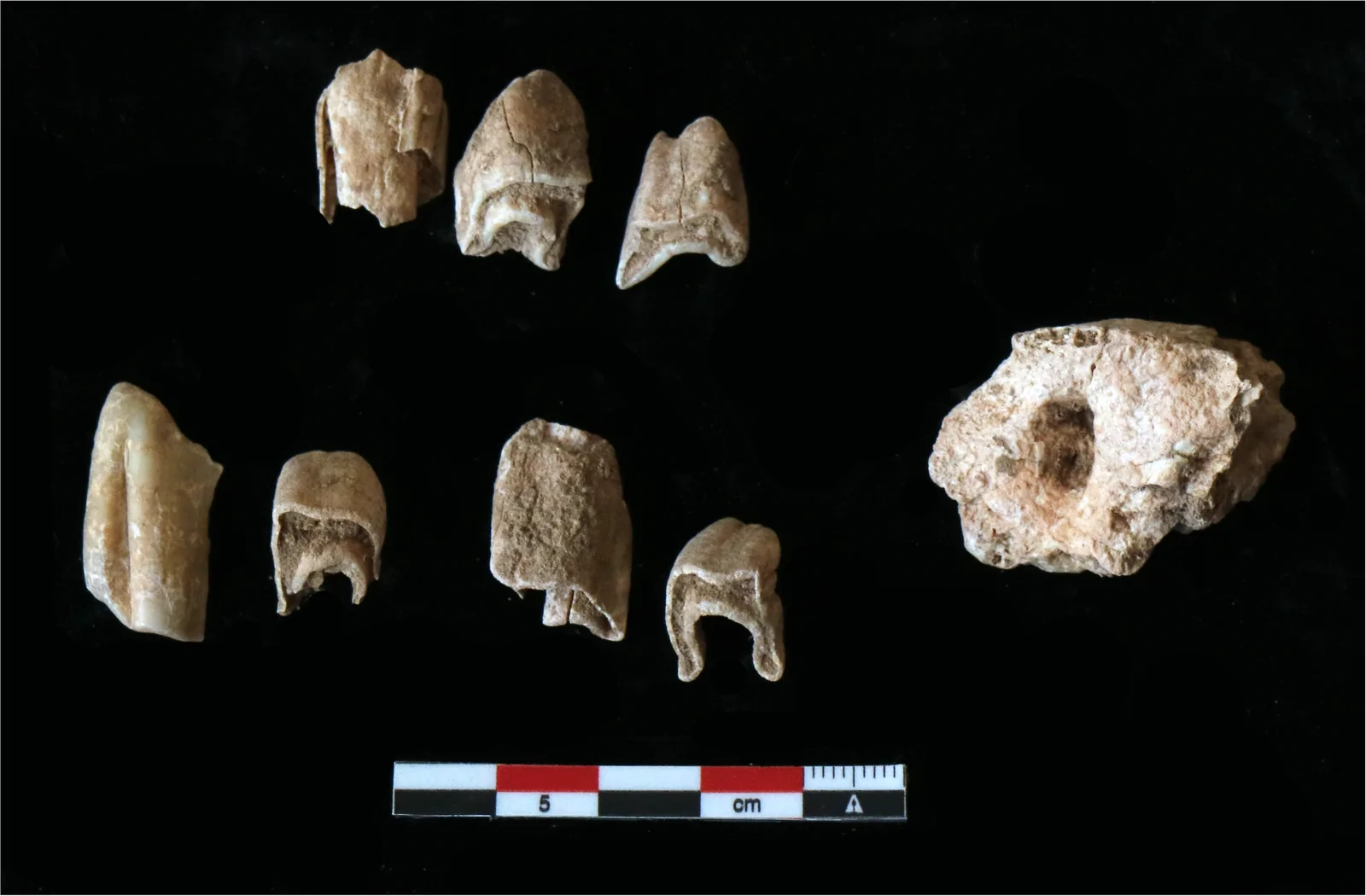
Faunal remains from excavations in mustatil IDIHA-F-0050739. The sample shows the anatomical representation documented in the mustatils of Al-Thamad area and the poor preservation of the remains. Left: the enamel portion of cattle maxillary teeth; right: cattle right petrous bone.

Excavations in IDIHA-F-0050739 revealed the largest corpus of remains, with 513 (270.2 g) faunal elements collected. Faunal remains were distributed in the sequence as follows: two remains in (001), eight in (002), 61 in (004) and 442 remains in (006). Preservation was poor with surface weathering observed, with tooth fragments predominating (64% of the total, with just the enamel portion preserved in most cases). Only petrous bones are preserved in their entirety (see Fig 25). In the assemblage 203 fragments could not be identified (39.6% of the total, 23.5% of the total weight). Of the remaining 310 fragments, most are bovine (261), numbering 50.9% of the total (191.8 g; 71% of the total weight). Of these bovine remains, 259 could be attributed to domestic cattle, corresponding to a Minimum Number of Individuals (MNI) of five, all represented in context (006). Small ruminants are less frequent (three bones, MNI 1). Thirty-six elements were highly fragmented and could only be attributed to ruminants; two remains to large ungulates; whilst eight bones belong to rodents.

In terms of anatomical representation, almost all the elements, are parts of the skull (15 petrous fragments, 63 cranial box fragments, 316 maxillary teeth fragments), except for the one small ruminant long bone fragment from (001) and the rodent remains from (004). No mandibular elements were identified (see Fig 25). Tooth and petrous bones are represented by both left and right sides. As for age, no decidual teeth were recorded, with 17 premolars present compatible with individuals older than 2–2.5 years based on tooth eruption (Silver 1963).

Most of the faunal material (86.2% of the assemblage) was derived from context (006). Anatomical representation is restricted to cranial elements, 64 cranial fragments, 15 petrous bones, and 310 teeth (169 securely identified as maxillary). The taxonomic spectrum comprises exclusively ruminants, predominantly cattle (244 remains), with small ruminants also present. Taken together, the marked concentration of material, its selective anatomical profile, and the stratigraphic integrity of the deposit indicate that the context (006) assemblage is best interpreted as an original and representative deposition.

The rodent remains comprise head elements, limb and spine elements, the latter in anatomical connection (altogether MNI 1). These remains were all found in (004) and were most probably intrusive.

### IDIHA-F-0051633

The sondage excavated in IDIHA-F-0051633 revealed 118 faunal remains, 57 of which were classified as indeterminate due to poor preservation (Table 2). The faunal remains were distributed as follows: seven in (002), 12 in (003), 47 in (004) and 50 in (005) and two in (006). The remains recovered from (005) are considered potentially contaminated by upper and surrounding contexts resulting from rain-water flow (see above) and will therefore be considered more cautiously. The state of preservation was poor, with 49.2% of the assemblage consisting in tooth enamel flakes characterised by surface weathering. In terms of taxonomic representation, 43 tooth fragments (36.4% of the total) can be attributed to cattle (MNI 1), with these present in all contexts. They are distributed as follows: one in (002), five in (003), 17 in (004), 19 in (005) and one in (006). Small ruminants (10 tooth fragments) are attested to in (002; one tooth fragment) and (004; nine fragments). Indetermined caprines (three tooth fragments, MNI 1) are also present in (005). Large ungulate bones (two cranial bones) were found in (003) and ruminant bones (two tooth fragments) in (004) and (006). All the identified ruminant remains consist of parts of the skull, the majority of which are tooth fragments (58), in addition to one fragment of a petrous bone, and four cranial fragments of indetermined taxa. One hare limb bone was found in (005), with the periosteal surface well preserved. Seventeen tooth fragments from (005) corresponding to a minimum of three teeth present grey enamel areas and cracks related to exposure to fire but not complete carbonization [12]. The most significant assemblage was recovered in the lower layers of the structure, (004) and (005). Finds from (004) consist of cattle remains, small ruminant and ruminant bones, with 10 maxillary teeth of both cattle, and small ruminants recovered.

### IDIHA-F-0050796

Only seven remains were recovered from the small sondage excavated in IDIHA-F-0050796 (Table 2). Four were classified as indetermined, with the remaining three fragments related to domestic cattle, recovered from (006). Almost all faunal remains were found in (006), with a single indetermined fragment recovered from (004).

In summary, if we exclude the 264 indetermined fragments, the spectrum is composed predominantly of bovine remains (82% of the total, 305 domestic cattle; two *Bos* sp. fragments, Table 2); with IDIHA-F-0050739 revealing an MNI of five from a single layer (006) in a small part of the chamber. Additionally, 16 bone fragments (3.3%) from two structures (IDIHA-F-0051633 and IDIHA-F-0050739) belong to the broad category of small ruminants, that includes indetermined caprines and gazelle. Four bones (1%) could be attributed to indetermined caprines. A further 38 ruminant bones were identified (10.2%), in addition to four large ungulate bones (1.1%), eight rodent bones (2.1%) and one hare bone from an unsafe context (less than 1%). In terms of anatomical representation, 365 out of 374 identified bones belong to the skull, these include five cranial bone fragments (1.3%), 10 petrous bones (2.7%), and 350 tooth fragments (93.6%) of which 207 could significantly be defined as belonging to the maxillary (55.3% of the identified total). No mandibular elements or horn cores were identified within these assemblages. Similarly, no cut, chop or percussion marks could be observed.

### Surface sampled faunal remains

Despite the methodological difficulties of using surface material discussed above, we have included the following information for data transparency and to provide contextual data for the excavated remains, to aid in the overall interpretative framework of the site. With this in mind, a total of 43 remains were surface collected from the chamber of seven mustatils: 16 samples were collected from IDIHA-F-0051657; 15 from IDIHA-F-0051636; five from IDIHA-F-0051658; three from IDIHA-F-0051664; two from IDIHA-F-0051665; one from IDIHA-F-0051659; and one from IDIHA-F-0051666. The quantification of these remains is detailed below in Table 2. In terms of preservation, all remains were fragmented, in most instances only the tooth enamel was preserved. Due to poor preservation, 29 remains (11.5 g) could not be identified and are excluded from the following counts.

Three mustatils (IDIHA-F-0051636; IDIHA-F-0051658; IDIHA-F-0051659) revealed samples dating to the 5^th^ millennium BCE. The 21 faunal remains from these structures included: three cattle teeth, two *Bos* sp. teeth, two large ungulate teeth, and 14 indetermined remains. Excluding indetermined fragments, all specimens are elements of the head, with four maxillary tooth fragments. Two fragments from IDIHA-F-0051636 and one from IDIHA-F-0051658 were also characterised by partial exposure to fire. The samples from IDIHA-F-0051657, which have been dated to the early 4^th^ millennium BCE, consist of a large ungulate long bone fragment, a small ruminant vertebra fragment, and 14 indetermined fragments. Exposure to fire was also observable on 10 remains, of which seven were partially carbonised. Finally, for the six undated surface samples from IDIHA-F-0051664, IDIHA-F-0051665 and IDIHA-F-0051666, fragments of three cattle maxillary teeth were present (IDIHA-F-0051664) and two small ruminant teeth (IDIHA-F-0051665, IDIHA-F-0051666). The three bones from IDIHA-F-0051664 were also marked by exposure to fire.

Overall, in the taxonomic spectrum, ruminants, namely bovines are the most documented taxa recorded through surface sampling (eight bovine bones in four mustatils, including six cattle bones in three mustatils). Small ruminants are also attested to in three of the sampled mustatils. The anatomical representation mostly consists of cranial elements (12 bones) with maxillary teeth well represented (in four mustatils, including the three-5th millennium BCE dated structures). No mandibular elements were identified in any of the sampled structures and exposure to fire was documented on the remains from four mustatils. This absence of mandibular elements broadly corresponds to the evidence recovered from excavated contexts.

### Contextual comparison with the mustatils of AlUla

To date, 14 mustatils have been excavated across AlUla County, with the results from 11 of these excavations published [4–6]. Thirteen mustatil chambers were fully excavated to ascertain the nature of the assemblages and their depositional context. All revealed broadly homogenous, highly selective and structured deposits of faunal remains, specifically the upper cranial elements and horns of wild and domestic ruminants. Although the faunal composition varied across the structures, with some marked by higher proportions of wild taxa, others were composed predominantly of cattle or caprines. All fourteen structures have also been dated to the late 6^th^ to mid 5^th^ millennia BCE [4–6]. Of note is the deliberate placement of cranial elements around upright stones, as well as the occurrence of small hearths within the chambers [4–6]. The limited, albeit diverse range of non-faunal materials deposited in the chambers was largely quotidian in nature, including projectile points (IDIHA-F-0011356 [6], chert flakes, and ground-stone objects (IDIHA-F-0011081) [5]. We have previously suggested these objects may reflect the conception of the chamber as some form of ritualised occupation unit or dwelling [6]. In terms of the courtyards, artefacts have rarely been found in this part of the structure, either through survey or excavation. As such our understanding of this part of the structure remains incomplete.

The highly selective and structured nature of the deposits has led us to the conclusion that these are ritual deposits and that at least in AlUla the mustatils most probably reflect a cohesive ritual concept and behaviour.

## Discussion

### Domesticates, cult, and the mustatils of the Harrat Khaybar

As this research is the first of its kind on the mustatils of the Harrat Khaybar, it provides new and important information regarding the phenomenon more broadly. Analysis of the excavated faunal remains revealed that all the excavated assemblages were composed almost exclusively of the upper cranial elements from horned ruminants, an anatomical data set that does not correspond with daily food refuse. Instead, this strong taxonomical and anatomical patterning appears to be the result of specific and deliberate anthropic selection. Importantly, the documented taxonomical and anatomical profiles correspond to the data from the mustatils of AlUla. As such, we would assert that both data sets can be interpreted as cultic/ritual deposits. Indeed, in AlUla all excavated mustatils have revealed an emphasis on the intentional deposition of horned ruminants, specifically portions of the upper skull and horns [4–6]. Based on this data, we would suggest that the Al-Thamad structures can be associated with a larger ritual ideology and belief system. The material obtained from the surface sampling programme is also of note. Despite its small size and the methodological concerns associated with using surface material, the data obtained during this study broadly corresponds with that recovered from the excavated structures and demonstrates that material associated with at least one phase of a mustatil’s use can be found in areas close to, and directly on the surface, or within the remains of these structures.

In terms of the ritual activity undertaken at Al-Thamad, analysis indicates that faunal remains were the key find associated with these structures, with no other artefact categories documented. Despite the limited size of the excavation areas in the mustatil (head) chambers, these first results are significant. Horned ruminants, predominantly cattle, were the primary taxa documented across all these features, for a total of 372 for the identifiable remains. Examples were identified in all excavated mustatils and in five of the seven sampled mustatils (Table 2). Non-ruminant taxa are represented only by remains of a rodent (eight bones, two in anatomical connection; MNI 1) and a hare (one bone; MNI 1). The rodent remains from IDIHA-F-0050739 were recovered from (004). Context (004) lies high in the stratigraphic sequence and is characterised by bioturbation and a markedly low number of finds compared to the main faunal assemblage located in (006). Considering the stratigraphic position of the finds within an open and bioturbated context, the anatomical connection of some examples, the low number of bones, and the absence of anthropic marks, it can be asserted that these remains are intrusive. Similarly, the hare long bone found in IDIHA-F-0051633 was recovered from a post-rain cleaning context. This context maybe considered not secure, due to potential contamination from water runoff. Given this stratigraphic uncertainty, the lack of anthropic marks, the better preservation of the periosteum (suggesting a different diagenetic history), and the natural distribution of the taxon, this specimen can also be considered intrusive. The other specimens from the same context are also excluded.

As for anatomy, the cranium is the most frequent skeletal element documented. Elements not pertaining to the cranium are extremely limited, in total only 12 specimens were recovered (1.7% of the assemblage; 6.3 g, 1.8% of the weight). Importantly, none derive from low or undisturbed contexts. Three long bone fragments of a small ruminant, large ungulate, and indetermined taxon (<2 g each) were recovered from the surface of three mustatils (IDIHA-F-0050739, IDIHA-F-0051657, IDIHA-F-0051658). The rest comprise seven of the above-mentioned rodent bones and the hare bone. Such isolated finds are insufficient to draw conclusions that challenge the ritual pattern observed to date in AlUla.

The features of the faunal assemblages suggest horned ruminants were intentionally selected for deposition in the mustatil chambers, with petrous portions, maxillary teeth, and tooth enamel flakes consistently found throughout each of the investigated structures. The rest of the skull is represented in a low percentage (10% of total). Considering that teeth and petrous bones are the densest and most robust portions of the skull, such an anatomical representation may be biased by preservation, with the archaeological assemblage therefore representing all that remains of what were once more complete skulls, skull portions or maxillaries. This is further suggested by the appearance of both left and right sides of these elements.

The differential robustness of skeletal elements, stemming from variation in density, mineral content, and tissue structure has a significant effect on archaeological assemblages [13]. This is directly observable in the field, where some bone elements fragment or even disintegrate during excavation. The variations of preservation on mustatil faunal assemblages is particularly evident in the case of teeth: composed of highly mineralised, dense tissues, they are especially resistant to diagenetic processes [13]. In the better-preserved mustatil assemblages from AlUla, teeth are found complete, retaining their original shape, with both enamel and dentine preserved [4,5]. In contrast, in the assemblages examined here, teeth are consistently fragmented and in 267 instances (66% of teeth) only enamel, the most mineralised of the dental tissues preserved [13,14]. Comparable effects of preservation were already observable in AlUla, in mustatils that were not protected by rock overhangs [6]. Differential preservation may also explain the absence of horncores at Al-Thamad. Horncores which have been primarily documented in mustatils located under rock overhangs in AlUla [4–6]. Whilst mustatils not situated in such locales present lower numbers (or no) horncore fragments [6]. At Al-Thamad the restricted extent of the excavation area precludes a secure assessment of the original representation of horns/horncores within the assemblage. Consequently, the possibility of their absence, potentially reflecting a distinct depositional practice cannot be excluded. However, considering the overall poor state of preservation, the anatomy of ruminant skulls and the comparative evidence from other mustatils, preservation bias provides the most plausible explanation for this absence. Conversely, the systematic absence of clearly identified mandibular teeth is notable across the excavated mustatils in both AlUla and Khaybar, given the robustness of dental tissues. Moreover, mandibles are separate skeletal elements attached to the cranium only by soft tissue subject to early decomposition. This suggests a consistent and deliberate behaviour, with mandibles excluded from the deposits, a facet also evident in the excavated AlUla assemblages [4–6].

The emphasis on domesticates, principally cattle (45.7% of total; Table 2), at the study site aligns well with the available data from AlUla. Across all excavated mustatils in AlUla, domestic species dominate the assemblages, with cattle preponderating in seven of the excavated mustatils [6], while higher percentages of domestic caprines, specifically goats, were found in two [4,6]. As previously observed for the AlUla mustatil fauna [6], questions arise regarding a possible correlation between the lower frequencies of caprine remains in mustatils lacking a protective overhang and the poorer state of preservation for such contexts, circumstances that likewise characterise the Al-Thamad assemblages. Here preservation bias, along with the small scale of the excavation areas, may have resulted in small ruminants being underrepresented. Regardless of species proportions, the Al-Thamad assemblages do not exclusively comprise cattle: small ruminants occur in at least five mustatils, and indetermined caprines are identified in two. These remains cannot be attributed to a specific species, as such wild ruminants cannot be excluded. Wild ruminants are attested to in three mustatil assemblages in AlUla but they consistently represent a low percentage of the deposit (0.2%−5%) [4–6]. However, considering domestic taxa vs wild taxa frequencies, the current dataset points to a preference for domesticates in the Khaybar area. Moreover, based on the current data it would appear that at Al-Thamad, as in AlUla, mustatil ritual activity was not focused on a single taxon. This pattern possibly reflects a mixed subsistence animal economy, which combined herding with hunting, alongside a multi-taxa herd management strategy (cattle, sheep and goat). Such subsistence regimes for the region’s ancient inhabitants are documented across both mustatil and domestic contexts [15]. This fusion of ritual and economic lifeways appears to be mirrored across the Arabian Peninsula during the Neolithic. One such example is the monumental stone platform at Dūmat al-Jandal, where small faunal deposits, comprising *Bos* sp. maxillary teeth as well as caprine metapodia, were recovered from contexts dating to the mid-6^th^ millennium BCE [16]. A further example is the Shi'b Kheshiya (Yemen) Cattle Skull Ring, dated to the mid-5^th^ millennium BCE, where 42 (extant) fresh cattle skulls were deliberately arranged in a circular installation following sacrifice and feasting [17–19]. The emphasis on domesticates and skulls, with the mandibles deliberately removed, is noteworthy. However, several factors, for instance the architectural use of the skulls and the sole presence of cattle, distinguish this monument from the ritual practices documented at mustatil sites [5; see below).

As observed thus far, there is a marked consistency in the ritual selection of the offerings deposited in the mustatils of both Al-Thamad and AlUla. Besides taxa and anatomy, a possible preference for adults may be tested through future excavations. While no clearly identifiable decidual teeth were recorded, permanent premolars were present. The porosity of the bones of juveniles may have biased the archaeological dataset, however, it must be pointed out that even the well-preserved assemblages in AlUla show a similar trend [4,5]. In terms of ritual practices, further questions remain on the details of the preparation of the anatomical portions for deposition, and on its timing and location. Indeed, despite mustatil assemblages being in some instances quantitatively significant (up to 67 individuals) [4], the faunal remains attested provide no evidence for full carcass processing and consumption [5]. In addition, no cut nor chop marks were observed in the Al-Thamad assemblages contrary to AlUla, where head/cheek skinning is documented [4–6].

Due to the sondage methodology, it is not possible to study the spatial distribution of the remains, the importance of which is documented in other mustatils in AlUla through specific arrangements of the offerings in the chamber and/or in front of betyls [4–6]. However, it is worth noting that an MNI of five cattle documented in (006) of IDIHA-F-0050739, a relatively small area (1.8 x 2.2 m) at the end of the structure, suggests a highly packed chamber, similar to IDIHA-0000687 [4].

Finally, the relationship between the treatments of offerings and the role of fire in the mustatil assemblages of AlUla and Khaybar remains unclear. In AlUla, fire traces were observed on the faunal remains from some mustatils, specifically on several of the bovine horns and teeth [4,5]. Additionally, small fires or burning material was often placed either in front of the central standing stone (betyl), where present, such as at IDIHA-F-0011081 and IDIHA-F-0011356, or in the corners of or around the edges of the chamber (IDIHA-F-0011356; IDIHA-0000687) [4–6]. As such, the ritual use of fire or burning as a possible component of the ritual activity undertaken across the wider mustatil tradition, and potentially for the *in-situ* offering acts cannot be excluded. At Al-Thamad, traces of burning were observed on faunal remains, but only from contexts that cannot be considered secure (post-rain context in mustatil IDIHA-F-0051633) or the surface. As such, at Al-Thamad the possibility that fire traces derive from non-ritual activities cannot be completely ruled out.

The consistency in these assemblages across both Khaybar and AlUla is crucial to expanding our understanding of the mustatil tradition. It suggests the mustatils may represent a cohesive cultic/ritual concept, which revolved around the offering/deposition of selected portions of horned ruminants. As such, mustatils may not only have been characterised by a homogeneity of architectural concept (rectangular form) but also that of practice and perhaps belief. Indeed, if these findings can be extrapolated across the more than 1600 examples currently known from northern Arabia, which span some 350,000 km^2^, this has far reaching implications for understanding the development and spread of early belief systems and ritual behaviours across Western Asia during the Early-to-Middle Holocene. To date, scholars are divided as to how to address the idea of belief, cult or indeed ‘religion’ (if there was in fact such a concept) in the Neolithic [20–26]. For instance, how and when do early ritual or cultic practices transform into more concrete or regulated ideological systems, and what are the broader socio-cultural connections between these two developments? Although the mustatils do not yet provide such an answer, the consistency of the tradition in both its material expression, and possibly its underlying ideology, may suggest an early solidification and spread of an organised, systematic belief system. A system that was most probably born out of the social and economic frameworks of the mobile peoples who inhabited these challenging environments, where cultural knowledge is inextricably tied to places within the landscape rather than small-scale materiality [27,28].

The identification and possible prominence of cattle and *Bos* sp. bones within these structures, further reinforces the cultural significance that cattle held for the inhabitants of the Harrat Khaybar and wider Northern Arabia during the Neolithic [4–6,16,29–32]. This significance is also reflected in the rock art record of the region, with cattle overwhelmingly represented [30,31,33,34], but it is important to note that no depictions of a mustatil have been identified. Prior to these results, it was hypothesised that more evidence for wild taxa may be expected in this region, particularly in light of the vast networks of ‘desert kites’ that dot the landscape [35,36]. In the absence of (published) contemporary domestic assemblages from the Harrat Khaybar, the identification of domesticates, like cattle, within the faunal assemblages of the mustatils suggests that some degree of cattle and caprine herding was undertaken across the difficult basalt terrain of the region [37]. This provides important insight into the economy of the Neolithic inhabitants of this region, highlighting the importance of domestic taxa for these communities. This evidence demonstrates that domestic cattle were present in the area from at least the 5^th^ millennium BCE, expanding the known geographic distribution of domestic cattle in Northern Arabia. The degree to which these ritually deposited cattle remains directly reflect the animal economic strategies employed by these populations remains unknown. In AlUla, the faunal assemblages from the mustatils differ markedly from domestic context. While domesticated caprines dominate domestic sites, the distinct composition within the mustatils implies that faunal requirements for ritual practices differed from everyday subsistence [6,15]. As a biological proxy for palaeoenvironmental reconstruction, the mustatil data from Al-Thamad also suggests that herding in the 5^th^ millennium BCE in Khaybar was viable in terms of pasture and of surface water sources. Indeed, the highly distinctive geological make-up of the Harrat Khaybar with its multi-order dendritic surface network of lava-flows allows for seasonal run-off to be funnelled into large wadis and *qa’*, which retain surface water for much longer and have accumulated deeper deposits of alluvium, making these areas very attractive for human habitation [38–40]. The faunal assemblages from Al-Thamad with their focus on cattle suggest such a hydrogeological system was still active during the mustatil period, despite aridification proxies recorded in other areas to the North [41].

### Architecture, chronology, and temporality of the mustatil tradition

The architectural diversity of form at the Al-Thamad site is also notable, with at least four distinct variants identified across the area. Whilst the overall form of the mustatil was maintained, these variations were all associated with the base of the structure, as well as the associated features. These distinctions in form contrast with the data obtained from AlUla, where an almost strict uniformity of architectural type was present across the county. Although some intra-site architectural distinctions are visible in AlUla [3,6], when mustatils were found clustered together, form was often identical. This is best exemplified at IDIHA-0011233, where seven almost identical mustatils were constructed a top of a mesa in the Sharaan Nature Reserve [6].

**Fig 26:**
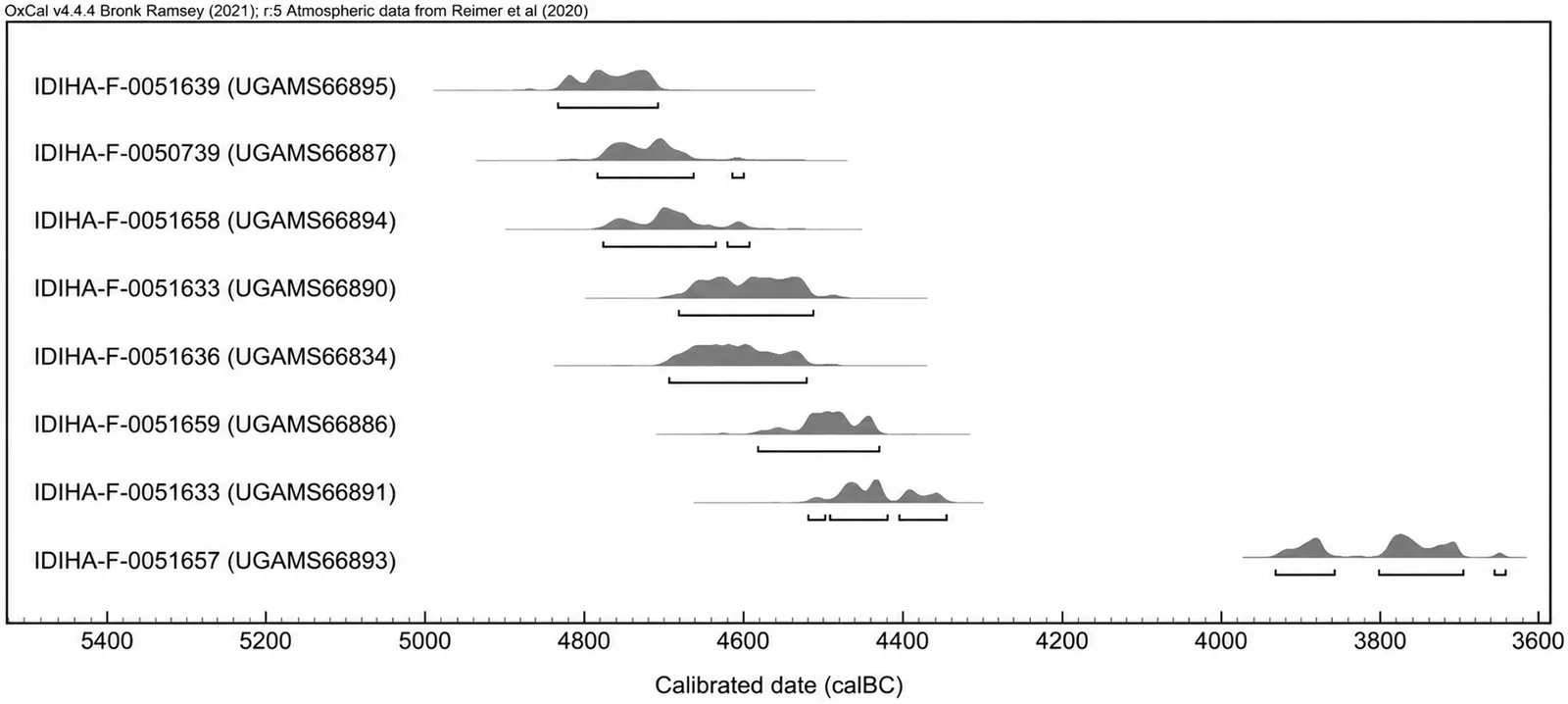
Radiocarbon data from all dated mustatils at Al-Thamad, Harrat Khaybar.

The presence of architecturally distinct but chronologically contemporaneous mustatils within the densely clustered examples at the Al-Thamad site is of note (Fig 1; Fig 26; Table 3). It suggests that changes in architectural form cannot be used as a chronological indicator within the mustatil tradition in the Harrat Khaybar. This is best exemplified by IDIHA-F-0050796 (Variant 3) and IDIHA-F-0051636 (Variant 4), which appear to have been in use at the same time but are marked by different architectural forms (Figure 26; Table 3). Parallels for this are also present in AlUla, albeit to a lesser degree, IDIHA-F-0011081 (Variant 1) and IDIHA-F-0051480 (Variant 1) are perhaps the best examples of this, with both dating to ca. 5200–5000 BCE. In this example from AlUla, each structure is marked by differences in form; IDIHA-F-0011081 is a complex mustatil with associated features, whilst IDIHA-F-0051480 a simple mustatil with no associated features [6]. As architectural distinction does not appear to be the result of chronology, other factors must be considered. For instance, one possibility is that these differences may be related to the groups who constructed them, with these distinctions in plan and design potentially indicating social or group differentiation within the broader congregation of a potentially disparate mobile population. Other possible factors may have revolved around seasonality or other unknown factors. Despite evidence for some architectural variability across the study site, there is no clear indication that these distinctions were associated with different cult practices. Rather, this variability is better understood as a product of differing contexts, scales, and resources. Each structure arises from its own unique context, while serving a shared ritual purpose. What changes is not the meaning, but the manifestation. Whether monumental or small-scale, both give architectural form to the same impulse: to create a sacred space.

**Table 3:** Radiocarbon data from all dated mustatils at Al-Thamad, Harrat Khaybar.

Lab ID	Sample ID	Material	Analysis	Feature	Age 14C (BP)	Calibrated Date BC 68.2% (1σ)	Calibrated Date BC 95.4% (2σ)
UGAMS66895	#1850	Tooth	Bioapatite	IDIHA-F-0051639	5910 ± 25	4828−4820 (0.094) 4798−4771 (0.368) 4764−4725 (0.538)	4839−4719 (1.000)
UGAMS66887	#1733	Tooth	Bioapatite	IDIHA-F-0050739	5860 ± 25	4783−4744 (0.571) 4731−4706 (0.429)	4796−4677 (0.985) 4631−4620 (0.015)
UGAMS66894	#1817	Tooth	Bioapatite	IDIHA-F-0051658	5840 ± 25	4779−4753 (0.223) 4728−4680 (0.770) 4627−4625 (0.007)	4788−4653 (0.910) 4638−4614 (0.090)
UGAMS68634	#1754	Tooth	Tooth	IDIHA-F-0051636	5780 ± 30	4689−4598 (0.0923) 4564−4555 (0.077)	4709−4547 (1.000)
UGAMS66890	#1822	Tooth	Bioapatite	IDIHA-F-0051633	5760 ± 25	4671−4665 (0.057) 4658−4635 (0.244) 4616−4550 (0.700)	4696−4539 (1.000)
UGAMS66886	#1821	Tooth	Bioapatite	IDIHA-F-0051659	5690 ± 25	4544−4492 (0.859) 4472−4460 (0.141)	4602−4562 (0.101) 4556−4453 (0.899)
UGAMS66891	#1755	Tooth	Bioapatite	IDIHA-F-0051633	5630 ± 25	4497−4443 (0.784) 4419−4400 (0.193) 4377−4375 (0.023)	4537−4515 (0.065) 4509−4439 (0.618) 4426−4365 (0.316)
UGAMS66893	#1820	Tooth	Bioapatite	IDIHA-F-0051657	5010 ± 25	3907−3878 (0.287) 3802−3760 (0.481) 3741−3712 (0.232)	3943−3863 (0.353) 3812−3706 (0.630) 3670−3660 (0.017)

This variation in architectural form at Al-Thamad is seen most clearly in the base of the structures and in the associated features. Firstly, the round cells (associated features) seen in AlUla are absent at the Al-Thamad site [3]. Instead, stone pillars, orthostats, and tunnels demarcate the beginning of the sacred space (see Fig 6, Fig 10, Fig 13). This is further exemplified in the base of IDIHA-F-0051636 (Variant 4), which was marked by its crescent shape, framing a row of standing stones that materially extend the boundary of this liminal space. One component of this is the practical limitations of the local basalt, which tends toward larger rounded boulders poorly suited to drystone masonry. Beyond this however, the tunnels associated with IDIHA-F-0050776 (Variant 2) suggests a more conceptual change in access to the structure, requiring individuals crawling on their hands and knees across the basalt. This would have been both difficult and performative and may have been deliberately designed to restrict the number of participants accessing the structure at any one time, or even to potentially elicit discomfort in the participants. If this was an aim of the tunnel system, it may suggest, at least in this instance, that a symbolic value may have been ascribed to participant restriction and physical discomfort. There are numerous ethnographic parallels for this, with physical discomfort and pain used to heighten the ritual experience, as well as a form of purification, or to increase group cooperation and connectivity [42–44]. The latter may relate to the mustatil phenomenon, this development being perhaps an extension of the monument building process. Conversely, this architectural complexity demonstrated in the mustatils at Al-Thamad may have been intended to incentivise navigating around and through open pathways, much like entry to the AlUla examples which were constructed around and behind long chains of connected cells, creating created narrow pathways to the entranceway, resulting in procession-like movement. Creating structured, ritualised movements both into and within the mustatil may have been intended to formalise a fundamental element of the Neolithic people’s lives: walking. This mirrors and extends their physical journey which commenced at their place of origin and ended with the construction of the monument and the dedication/deposition of the faunal elements. Importantly, these locales are rarely found in association with contemporary occupation and are in fact often at significant distance from substantive occupation sites. Due to their often-remote locations, the construction and use of a mustatil was most likely a socially and culturally driven element within the wider mobility pattern practiced by these people, requiring a ritualised journey to a certain location, for specific ritual and social activities [27,28,45–47].

The tight clustering of the radiocarbon data from the Al-Thamad area also suggests that specific sites across northern Arabia were the focus of special and repeated use, with these sites potentially functioning as a ‘central place’ in the Neolithic landscape [26]. That these sites rather than those of daily habitation appear to have been at the heart of this collective activity and monument building is of note. It is possible that there was a symbolic importance to the physical positioning of these monuments, one that was derived from the value of the offerings themselves – domestic taxa and other horned ruminants. The positioning of these monuments in areas of higher vegetation and surface water, speaks to a prioritisation of the key requirements needed for the herding of animals. As such, the needs of the herd and control of pastoral resources may have dictated the location and placement of the mustatils [48]. Whilst this is evident in AlUla where the mustatils are regularly placed near strategically located, seasonally available water sources [5], this aspect appears to be heightened in the Harrat Khaybar; where water and rich soils are more abundant, longer-lived, and closer to each other. This positioning may therefore account for the lack of consistency in orientation, with this not a principal consideration, with location rather than orientation prioritised as a principal element of the tradition.

The later date from IDIHA-F-0051657 (Variant 1) is also of note. This sample (UGAMS66893) was not from cattle but a small ruminant, obtained from within the chamber of this structure. Although UGAMS66893 cannot be identified to species level, and wild taxa cannot be ruled out, this example may represent a later ritual deposit. Importantly, this sample dates to the early 4^th^ millennium BCE, a period which postdates the widespread use of the mustatil tradition, based on current knowledge. There is precedent for this in excavations at IDIHA-F-0011356 which revealed an early 4^th^ millennium BCE deposit [6]. The rear chamber of this feature had been modified, with a deposit of domestic sheep identified. Two samples from this feature were radiocarbon dated, the first had a 2σ spread of 3946−3714 cal. BC, and the second 3938−3658 cal. BC [6]. The elements deposited also differed, with axial elements and limbs, rather than upper cranial portions identified. These examples from AlUla and Khaybar may indicate a later phase of dedication or use, that post-dates the initial flourishing of the tradition and the broader Neolithic horizon of northern Arabia [15]. This also correlates with an emerging pattern of potential post-Neolithic memorialisation of these features with both deposits of faunal and human remains [3,6].

**Fig 27:**
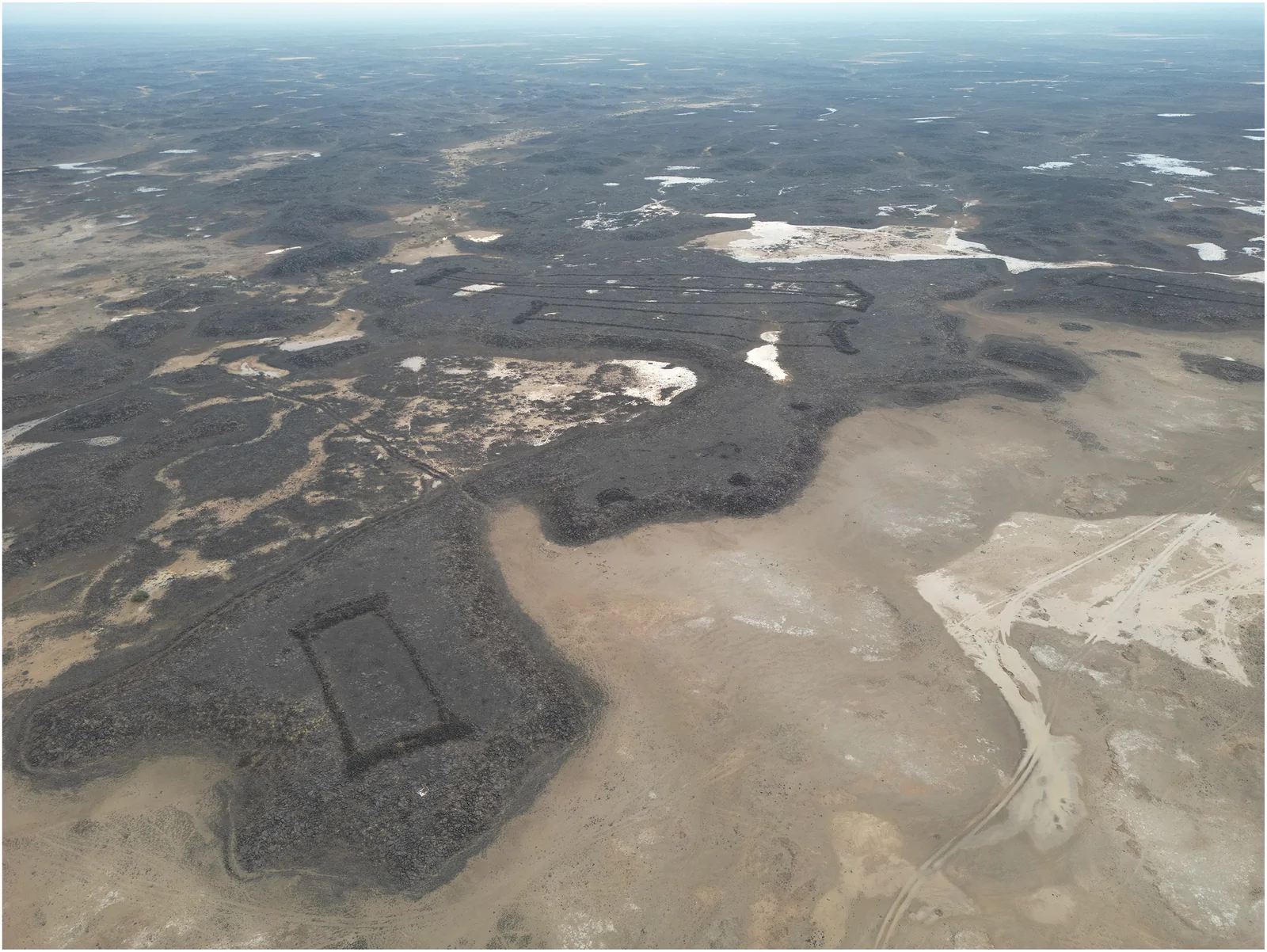
Positioning of mustatils above various *qa’* (after rain) at the study site, photo orientated to the south-east.

Despite these uncertainties, when combined, the dates from AlUla and Khaybar suggest a much longer chronological span for the mustatil tradition than had previously been supposed [6], one totalling almost 1000 years. Based on these dates and those previously published from AlUla it can be hypothesised that the tradition can be dated to ca. 5300−4400 BC. If accurate, such a date range would make the mustatil tradition one of the earliest, long-lived, and potentially cohesive belief/ritual-systems currently know. Further to this, that all the mustatils investigated at Al-Thamad appear to date chronologically later, in the 5^th^ millennium BC, than the published examples from AlUla, is significant [4–6]. Indeed, the earliest examples from AlUla, IDIHA-F-0011081, IDIHA-F-0011356 and IDIHA-F-0051480, predate the mustatils discussed here by more than 400−500 years [6]. As such, based on the currently available evidence it can be hypothesised that the mustatil tradition may have appeared earlier in AlUla, before spreading southward and eastward to the Harrat Khaybar and perhaps beyond. We had thought, that based on the density of the mustatils in this area (more than 400), that the Harrat Khaybar may have been the epicentre or origin of this tradition, but based on the current data, that may not be the case. Although we do not yet know the precise origin of this tradition, it is possible that it finds its genesis further to the northwest in either AlUla or just beyond. As such, this later date range may suggest that the mustatil tradition may have taken several hundred years, after its inception, to penetrate the broader Harrat Khaybar. Similarly, our extensive (unpublished) mustatil radiocarbon dataset from AlUla (20 structures; 62 radiocarbon assays) has revealed a clustering of dates between ca. 5300−4700 BCE, with only a single post-4700 BCE outlier. The almost complete absence of later 5^th^ millennium BCE radiocarbon assays from the AlUla mustatils is of note. Does this clustering of dates represent a ritual/cultic shift in focus from AlUla to the Harrat Khaybar? If so, is this potentially in response to northwestern Arabia’s increasingly arid environment [41,49,50]? In AlUla, almost all the mustatils are positioned in the north and east of the county, such as the sandstone Badlands and in the Gharameel/Hamra Desert, areas today that are hyper-arid and unable to sustain cattle herding, as they lack the long-term surface water and vegetation needed for such a resource intensive taxon [51,52]. Therefore, if these areas did in fact become too difficult to sustainably graze herds of cattle or even caprines, did the geographic ritual focus of these groups shift southeast to the Harrat Khaybar, where areas of greater surface water and vegetation could be found thanks to the hydrogeological specificities of the area [39,40]? Spatially, the vast majority of the Harrat Khaybar’s mustatils are clustered above and around such areas, including at Al-Thamad (Figure 27). The importance and proximity to water is also supported by Hatton et al.’s [48] recent spatial study of the mustatils around the southern and western margins of the Nefud Desert. However, despite the clear importance of water to the placement and positioning of mustatils, it should be noted that this discussion relies on a very small sample of the 1600 + identified mustatils in northern Arabia. As such, although none of these scenarios can yet be confirmed, they offer intriguing possibilities for exploring the beginnings and movement of this phenomena across the cultural and settlement landscape of the north Arabian Neolithic.

### The Mustatils and the Neolithic ‘Cattle Cults’ of Arabia and the Sahara

Finally, the data presented above, when considered alongside recent research on the mustatils of AlUla, permits several preliminary observations regarding the broader phenomenon of the so-called ‘cattle cults’ of the Arabian Neolithic and Saharan Africa. While a more comprehensive, thematic treatment of this topic is currently in preparation by the authors, the discussion below allows for an initial framing of what we believe are they key issues and interpretive directions of this research in the context of the mustatil phenomenon, it is not designed to be an in depth comprehensive discussion of these traditions.

In our initial paper on the mustatils [3], we speculated that the predominance of cattle remains in IDIHA-F-0011081 (IDIHA-0008222) was indicative of the existence of a Neolithic north Arabian ‘cattle cult’, that is, a belief system centred on a cattle-related deity or one in which cattle constituted the principal focus of cultic activity [53–59]. On the basis of further excavations [6], this interpretation must be revised. Although the deposits within most of the mustatils demonstrate a preference for cattle, they were not the exclusive focus of these depositions. Rather, the emphasis appears to have been on horned ruminants more broadly, of which cattle are the most frequently represented species [4,6]. Instead, the deposition of the ruminant horns and skulls can be understood as an offering to an unknown deity/entity or purpose, rather than the central focus of the activity being undertaken; the deposits form an ‘offering’ rather than representing a ‘divinity’ [57,60]. This would appear to contrast with other ‘cattle cults’ from Neolithic Arabia and the Sahara, most notably with that of Shi’b Kheshiya [17–19,61,62].

Excavations at Shi’b Kheshiya revealed a ring of 42 (extant) cattle skulls positioned nose down into the ground and arranged around a central standing skull [18,19]. These remains were also found in association with a small stone platform and standing stones [17,18]. Many parallels can be observed between this structure and the mustatil tradition, i.e., the use of standing stones; the emphasis on cattle; the absence of mandibular elements; as well as the likely inclusion of feasting, and the potential that both features functioned as markers of territoriality and mechanisms for cementing social cohesion [5]. However, we would assert that significant functional and perhaps ideological distinctions between the two phenomena are observable. Firstly, at Shi’b Kheshiya the exclusive use of cattle skulls, and placement of a central standing skull, implies that the cattle themselves were key to the activity being undertaken, either as a representation/proxy for an unknown deity or perhaps as the central focus. This does not appear to be the case with the mustatils, as the horns and upper cranial elements of different (multiple) taxa (both wild and domestic) were deposited, with no species exclusivity identifiable.

Secondly, the sheer number mustatils differs from that of the unique instance of cultic practice at Shi’b Kheshiya. Whilst only a single cattle skull ring has been identified, to date, more than 1600 mustatils have been recorded across northern Arabia, spanning almost a 1000-years, indicating that the mustatils were part of a wider long-term framework of practice or belief [6]. This cohesion is further suggested by the overall architectural homogeneity of the mustatils, with the key architectural elements repeated over a vast geographic range. Finally, the monumentality and the construction materials of the two traditions differ considerably. Mustatils range in size from 20–620 m in length [3], with an average length of more than 100 m, incorporating thousands of tonnes of stone, all of which suggests that collective, large-scale monument building and marking the landscape was as important as the consumption and ritual deposition of the faunal elements. This contrasts with the comparative small scale of the Shi’b Kheshiya (1.75 m wide) skull ring and its construction from purely organic/perishable materials [61].

Clear distinctions can likewise be drawn between the mustatils and the so-called Saharan ‘African Cattle Complex’ [60]. The latter encompasses a diverse array of practices spanning a much longer chronological range than the mustatils, from ca. 4400–300 BCE [57,62–64]. These practices include cattle burials in which fully articulated or disarticulated, but anthropogenically modified, animals were interred within stone tumuli, such as those documented at Nabta Playa [59]. Elsewhere, cattle occur as components of human burials (possibly as food offerings), for example at Afunfun [57] and, more frequently, in the Nile Valley [57,59]; or as foundation offerings beneath stone monuments in the Libyan Sahara [60] or as evidence for feasting and rain making activities [62]. In addition, undated rock art images depicting cattle sacrifice have also been identified in the Messak mountain ranges of Libya [60,62]. Such diversity of forms, combined with the long chronological span of these practices, complicates efforts to determine their precise meanings and precludes an assumption of an underlying belief system. As a result, there remains ongoing scholarly debate as to whether cattle were regarded as ‘divine’, or treated as offerings, or even deposited in an ad hoc, non-ritual manner [57]. This ambiguity contrasts with the consistency within the mustatil rituals, where the upper cranial remains of horned ruminants appear to have been deliberately deposited as cultic offerings rather than the focus of dedication or ‘worship’. Other distinctions between the Saharan examples and the mustatils can be seen in the ages of the animals deposited (sub-adult to adult) and in the overall composition of these deposits [64]. The Saharan examples include the full age spectra of animals and all parts of the carcass in various states [57], whilst the faunal deposits recovered from the mustatils is highly specific, with only upper cranial elements of older animals deposited. Furthermore, a strong patterning is evident within the Saharan deposits, with the animals often placed on their right side and oriented east-west [57]; no comparable consistency of orientation is observed within the mustatils.

Despite architectural and possible cosmological differences between the mustatils, Shiʿb Kheshiya, and the so-called ‘African Cattle Complex’, all three are characterised by the shared prominence of cattle symbolism, a feature common to pastoral societies both past and present [46,62,63]. This emphasis may reflect how each community responded to episodes of climatic fluctuation, resource stress, and/or landscape transformation [62]. In such contexts, ritual practice likely functioned to mediate uncertainty and to reinforce collective strategies of social cohesion during periods of change; conditions that appear to have characterised much of north Arabia at this time. What differentiates these traditions, however, is their scale and apparent cohesion. The mustatils, with their greater consistency of form and composition, may suggest a more unified, large-scale cultic concept, one not yet evident in either southern Arabia or Saharan Africa.

## Conclusion

Whilst archaeological studies of the mustatil tradition have been limited to date, we are rapidly amassing a robust dataset for the chronology and function of these structures. The data presented in this paper provides the first evidence for the date and composition of the mustatil tradition outside of AlUla County [4–6]. This research may indicate that the mustatils were part of a single, cohesive tradition, focused on the ritual offering of the upper cranial elements of horned taxa, primarily domesticates. Based on this data, we can begin to hypothesise that the mustatils represent a homogenous ritual or cultic tradition, which extended over a large geographic area, some 350,000 km^2^. Furthermore, dates gathered from the mustatils of the Harrat Khaybar demonstrate a longer-lived tradition than previously thought, at least 1000 years of consistent practice. The later dates found in this assemblage also indicate a shift in the intensity of the tradition from AlUla to Khaybar, raising interesting questions regarding the nature of Neolithic mobility, and adaptive strategies employed by these communities in the face of a changing climate and environment [65]. This has wide-reaching implications for the study not only of the Arabian Neolithic but also the origin, development, and spread of early belief systems across Western Asia during an era of rapid and widespread cultural, economic and environmental change.
